# Learning and strategic imitation in modelling farmers’ dynamic decisions on bovine viral diarrhoea vaccination

**DOI:** 10.1186/s13567-022-01112-2

**Published:** 2022-12-02

**Authors:** Lina Cristancho-Fajardo, Elisabeta Vergu, Gaël Beaunée, Sandie Arnoux, Pauline Ezanno

**Affiliations:** 1 Université Paris-Saclay, INRAE, MaIAGE, Jouy-en-Josas, France; 2 INRAE, Oniris, BIOEPAR, Nantes, France

**Keywords:** Endemic disease, control, vaccination, BVD, farmers behaviour

## Abstract

Considering human decision-making is essential for understanding the mechanisms underlying the propagation of real-life diseases. We present an extension of a model for pathogen spread that considers farmers’ dynamic decision-making regarding the adoption of a control measure in their own herd. Farmers can take into account the decisions and observed costs of their trade partners or of their geographic neighbours. The model and construction of such costs are adapted to the case of bovine viral diarrhoea, for which an individual-based stochastic model is considered. Simulation results suggest that obtaining information from geographic neighbours might lead to a better control of bovine viral diarrhoea than considering information from trade partners. In particular, using information from all geographic neighbours at each decision time seems to be more beneficial than considering only the information from one geographic neighbour or trade partner at each time. This study highlights the central role that social dynamics among farmers can take in the spread and control of bovine viral diarrhoea, providing insights into how public policy efforts could be targeted in order to increase voluntary vaccination uptake against this disease in endemic areas.

**Supplementary Information:**

The online version contains supplementary material available at 10.1186/s13567-022-01112-2.

## Introduction

Accounting for farmers’ decisions regarding health-related practices is key to better understand and predict livestock disease spread at a large scale. Recent studies [1–3] have approached this topic—focusing on vaccination—mostly from an econometric perspective, where vaccination decisions are usually taken once, at the start of an epidemic outbreak. However, the dynamic influences that farmers may have on each other regarding control-related practices have rarely been considered. In [4], an integrative model was proposed to account for farmers’ dynamic decision-making process regarding the adoption of a binary control measure. In particular, phenomena such as learning and strategic imitation were considered. However, this model lied on a theoretical SIR infection component, and assumed that farmers share decision-related information only through the trade network across which the disease spreads. This paper presents an extension of such a model with an application to a specific disease: bovine viral diarrhoea (BVD).

BVD is a viral bovine disease that leads to economic losses and to a reduction in animal well-being (abortion, calving delays, and mortality) worldwide [5, 6]. In particular, the infection of females during mid-gestation can lead to the birth of persistently infected (PI) calves [7]. PI animals generally have a reduced lifespan [8] and are highly infectious during their entire lives. Therefore, they not only threaten immunity in the herd they belong to—by causing new infections within it—but also spread the infection to neighbouring herds through pasture contacts and trade movements [9, 10]. According to the simulation results from a previous modelling study [11], a combination of testing and mandatory vaccination may be able to eradicate BVD. However, since this combined strategy has not been shown to be economically cost-effective, control is often left to farmers voluntary vaccination. This can be a viable alternative for limiting new infections, if vaccination is performed regularly [11, 12]. In particular, European countries use vaccines that can prevent vertical transmission if administered before gestation [12]. For such vaccines, and for some standard breeding systems, it has been shown that vaccination might be an economically interesting measure for controlling BVD virus spread [13].

The aims of the present study were two-fold. First, we sought to extend the decision-making mechanism of the general model initially proposed in [4], by considering several scenarios with respect to how information is shared among farmers. In particular, we assumed that information is shared via “neighbourhoods”, defined on the basis of the animal trade network or the geographic proximity of herds. Second, we aimed to adapt the generic model to focus specifically on BVD, which involved modifying the definition of the costs on which farmers base their decisions.

The paper is structured as follows. First, we present the BVD model and the data used to construct it. Then, we detail farmers decision-making regarding vaccination by presenting the extensions made to the original general model. Following this, we define simulation settings, and present the associated results concerning BVD virus spread and the decision dynamics. Finally, we present a discussion on the basis of these results, focusing on a comparison between cases where decision-related information is shared among geographic neighbours, and those where it is shared among trade partners.

## Materials and methods

Our study is structured by two main components: one for the epidemiological-demographic dynamics of BVD, and one for farmers dynamic decision-making regarding the adoption of vaccines in their own herd. In the following, we describe each of these two components, together with the simulation setting used in our study.

### Epidemiological-demographic model for BVD

For the first component, we built a BVD discrete-time stochastic individual-based model that accounts both for within-herd and between-herd infection dynamics. More specifically, the introduction of the virus into a BVD-free herd occurs either through the purchase of infected animals or through close contacts with infected geographic neighbours during the grazing season.

The BVD stochastic individual-based model represents each animal and its main characteristics: sex (male, female), parity (P0, P1, P2, P3, P4, P5), age (in days), life-cycle, and health-state. Parity P0 corresponds to nulliparous animals (i.e. which have never given birth). This includes fattened females or females to be bred, and males. Parity P1 corresponds to females that have had a first calving, and so on up to Parity P5, which corresponds to females that have calved five times or more (i.e., at calving, the parity of the breeding female increases by one unless it is already P5). This model enables the representation of most herd types: breeding herds, fattening units of calves, and young beef bulls.

For animal movements, observed trade data were plugged-in, so this component of the model is deterministic. For the other components, in particular the life-cycle and health-state dynamics, certain transitions were assumed to be deterministic while others were considered to be stochastic. The parameters that determine transitions were either calibrated on data or fixed according to existing literature or expert knowledge.

### Data description

We considered data for a five-year period (January 2009–December 2013) relative to holdings in Saône-et-Loire, a French department densely populated with Charolais, a breed of beef cattle. It was shown in [13] that BVD virus spread can have a strong economic impact on the Charolais breeding system, and that -when compared to other breeding systems- the Charolais one is particularly sensitive to vaccination against BVD. Hence, this region represents a relevant case for the implementation of our study.

The data were obtained from the French cattle identification database (FCID), which records the complete life history of each bovine animal from birth to death. In particular, this includes all movements between holdings. For each animal, the available data include country code, national identification number, sex, breed, birth date, date of entry/exit into/from each holding it belonged to, and cause (entry: birth or purchase, exit: death or sale). In this study, we focused on herds owning beef animals. Indeed, given the low levels of dairy animals in the area of study, accounting for the latter would unnecessarily complicate the BVD model and parameter calibration.

In total, in the studied area, there were 4978 active herds (i.e., that had a positive number of beef animals at least once during the five-year period). Only holdings whose annual average number of beef animals was at least 30 for at least one of the 5 years were included (irrespective of whether they exchanged animals or not). This was done in order to focus on a set of holdings that could play a role in pathogen spread, and to reduce the computational complexity of the model. Additionally, 42 holdings were excluded either because they were of size zero on January 1^st^ 2009 and could therefore not be initialised for simulation, or because they had no births or gestating animals during the study period, which precluded computing certain herd-specific parameters.

In total, the study metapopulation consisted of 3416 herds (of the 4978 active herds). The percentage of the overall trade in animals linked to excluded holdings over the whole study period was only 4.2%.

Table 1 contains a summary of the distribution of movements between groups of herds, according to whether they belonged to the study metapopulation, to Saône-et-Loire but not to the metapopulation, to the rest of France, or to an unknown location. The herd size distribution in Saône-et-Loire within and outside the metapopulation can be found in Additional file 1A.

**Table 1:** **Distribution of animal trade movements according to the origin/destination group.**

Origin/Destination	Metapop (%)	Small (%)	Outside (%)	Undetermined^a^ (%)
Metapop	15	3.0	16.6	46.2
Small	1.7	0.2	0.8	1.6
Outside	12.2	1.5	0.0	0.0
Undetermined	1.0	0.1	0.0	0.0

Percentage of the total movements concerning herds in Saône-et-Loire from 2009 to 2013: the group of herds included in the study metapopulation (“metapop”), the group of excluded herds from Saône-et-Loire (“small”), and the group of herds in France but outside Saône-et-Loire (“outside”). Movements whose origin/destination is not known are grouped as “undetermined”.

^a^Probably corresponds to movements to other countries.

**Figure 1:**
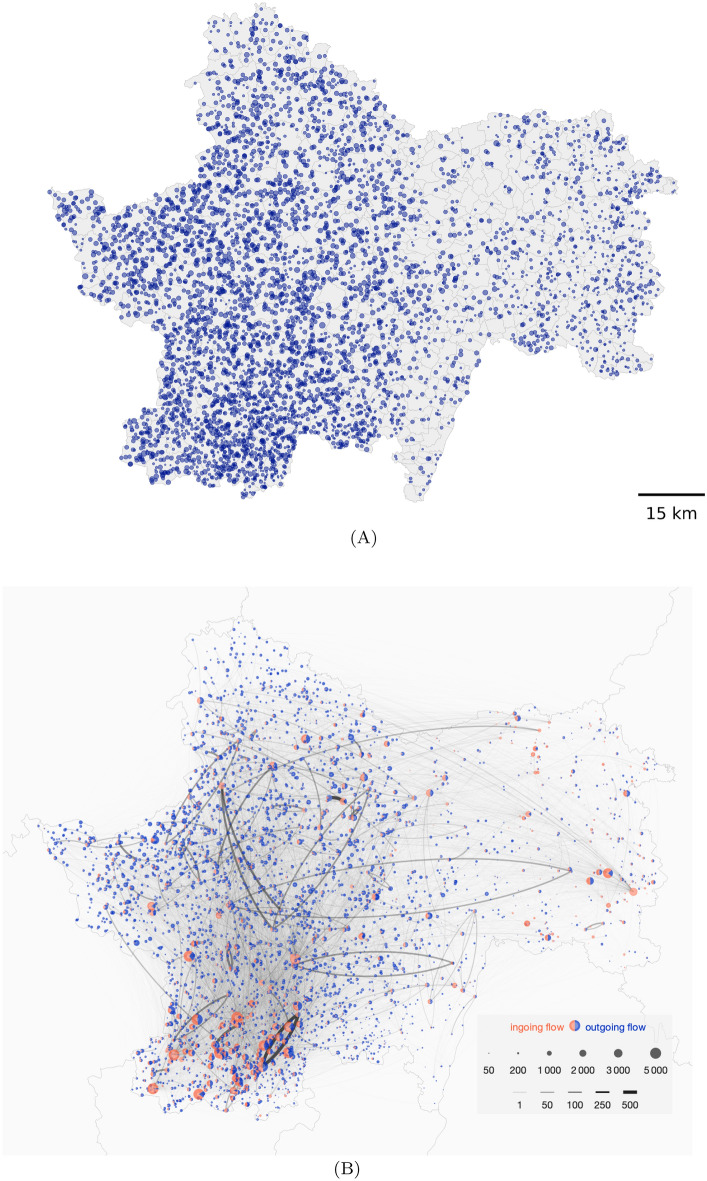
**Geographic location of exchanging cattle holdings.** The location of each holding was randomly assigned according to the coordinates of the administrative area the holding belongs to (on average ~6.97 holdings per administrative area in Saône-et-Loire. There are administrative 565 areas). The size of a point represents its average (annual) population. **A** Cattle holdings in Saône-et-Loire from 2009 to 2013 (4978 holdings). **B** Cattle holdings in the study metapopulation (3416 holdings). Colours of nodes in the network represent the mean (annual) share of purchasing (orange) and selling (blue). The links represent the existence (at least once within the period) of animal trade movements between two holdings in the dataset, with their width indicative of the observed trading volume along this link.

Figure 1A represents all holdings in Saône-et-Loire that were active at some point during the five-year study (4978 herds), highlighting a certain heterogeneity in the spatial density of the herds. The exact location of each holding was randomly assigned inside its administrative area (565 in total). Figure 1B represents the geographic location of holdings in the study metapopulation and their trade flows over the study period, also indicating a high spatial heterogeneity in trade patterns. Three geographic groups of holdings can be identified as being the most active in cattle exchange (in particular with a higher share of purchasing): one group in the south-west zone, one in the north, and one in the east. The first and second groups were more interconnected (more links, some with high trading volume) with respect to the connections with the group in the east, as herds in the latter group were not as connected within their group, nor with the rest of the metapopulation, except for some long-distance connections.

### Handling trade movements based on data

At each time step and for each herd, animals to be sold are chosen uniformly at random among the animals of the herd that have the required characteristics (sex, parity, life-cycle, age, date of entry into the life-cycle) according to cattle exchange data. If the destination herd is within the metapopulation, animals are sent to this herd. Otherwise, they are simply removed from the herd of origin. If the herd of origin does not have enough animals to sell with the right specifications, such animals are created in the destination herd. This also happens for animals coming from herds which are not within the metapopulation, but going to herds that are within the metapopulation. In both cases, the incoming animals are assumed to be susceptible, so the associated infection risk is null. Movements whose origin and destination are both outside the metapopulation are ignored.

## Life-cycle and health-state dynamics

The two main components of our BVD model concern life-cycle dynamics and health-state dynamics. Indeed, BVD transmission is mainly shaped by: (i) the contact structure between young animals and breeding females, (ii) the possibility of both horizontal and vertical transmission, and (iii) the fast renewal of the population (which makes it difficult to reach herd-immunity). In the following, we present the main characteristics of such dynamics, whose details can be found in Additional file 1.

**Figure 2:**
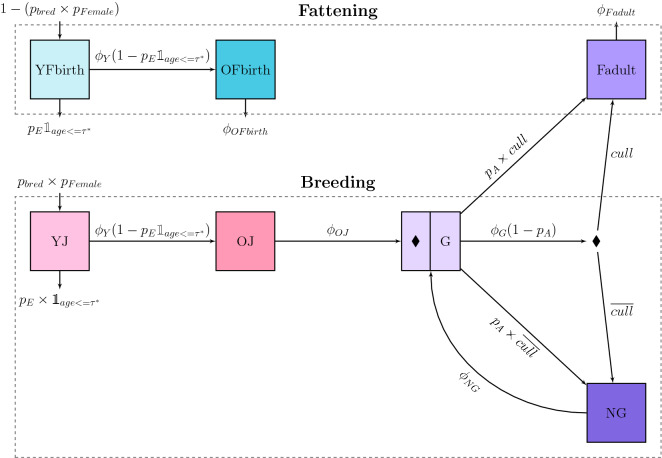
**Life-cycle dynamics.** Arrows represent transitions between life-cycle compartments described in Table 2 (*YJ* young juveniles, *OJ* old juveniles, *G* gestating females, *NG* non-gestating females, *Fadult* fattened when adult, *YFbirth* young fattened from birth, *OFbirth* old fattened from birth). The coefficients on the arrows are either transition rates or transition probabilities. A diamond in G indicates that when entering this state, it is determined whether the female will be culled after gestation or not (and the initial health-state of the future calf is defined). The other diamond indicates that after calving, the parity of a breeding female increases by one. See Tables 3, 4 and Additional file 1 for parameter definitions.

In our BVD model we considered seven possible life-cycle categories: YFbirth, OFbirth, YJ, OJ, G, NG and Fadult (defined in Table 2). Figure 2 shows a schematic of life-cycle dynamics. We first distinguished between fattened animals (males, and females not kept for breeding or fattened before culling), and females kept for breeding. Indeed, this distinction, as well as age and other demographic factors (gestating, not gestating, or to be culled) greatly impacts contact structure and BVD transmission. We note that it is possible to account for more fine-grained detail in an animal life-cycle, but the dynamics we have considered here allow the model to be as simple yet realistic as possible.

**Table 2:** **Life-cycle categories**.

Name	Description
YFbirth	Young animals Fattened from Birth, i.e., animals under 6 months of age that will never calve (male calves and female calves not kept for breeding)
OFbirth	Old animals Fattened from Birth, i.e., animals over 6 months of age that will never calve (old male and female animals not kept for breeding)
YJ	Young Juveniles. Female calves kept for breeding, under 6 months of age
OJ	Old Juveniles. Female animals kept for breeding, between 6 months of age and the beginning of the first pregnancy
G	Gestating female
NG	Non-Gestating female, i.e., in the period between calving and the start of the next pregnancy
Fadult	Fattened Adult female, i.e., in the period between the last calving and culling

**Table 3:** **Summary statistics of the herd-specific parameters of the model from the FCID**.

Parameter	Definition	Mean	10^th^	Median	90^th^
${p}_{Female}$	Proportion of females born in the herd	0.49	0.25	0.49	0.71
${p}_{bred}$	Proportion of female calves going to breeding	0.5	0	0.51	1
${\phi }_{J}^{-1}$	Total duration for animals in YJ and OJ	795.6	715.2	792	871
${\phi }_{NG}^{-1}$	Duration for animals in NG	133.8	84.6	121.7	190.3
${\phi }_{Fadult}^{-1}$	Duration for animals in Fadult	323.5	222.3	314.6	434.7
${\left({\phi }_{Fbirth}^{Male}\right)}^{-1}$	Total duration for male animals in YFbirth and OFbirth	532.7	160	486.3	956.4
${\left({\phi }_{Fbirth}^{Female}\right)}^{-1}$	Total duration for female animals in YFbirth and OFbirth	798.6	289.4	867	1100.5
${p}_{cull}^{P0}$	Proportion of females of parity *P*0 culled	0.25	0	0.2	0.54
${p}_{cull}^{P1}$	Proportion of females of parity *P*1 culled	0.23	0	0.2	0.5
${p}_{cull}^{P2}$	Proportion of females of parity *P*2 culled	0.22	0	0.18	0.5
${p}_{cull}^{P3}$	Proportion of females of parity *P*3 culled	0.23	0	0.2	0.5
${p}_{cull}^{P4}$	Proportion of females of parity *P*4 culled	0.25	0	0.2	0.57
${p}_{cull}^{P5}$	Proportion of females of parity *P*5 culled	0.38	0.12	0.34	0.66
${p}_{E}$	Proportion of calves dying before 21 days of age (over the whole 21-day period)	0.08	0	0.04	0.18

All durations are in days.

All 3416 herds in the study metapopulation are considered.

Missing values were replaced by the average over all months/years of a herd, or if the herd had no value for that parameter, they were replaced by the average over all herds for the month/year.

**Table 4:** **Life-cycle and health-state parameters of the model**.

Parameter	Description	Value	Sources
${\phi }_{Y}^{-1}$	Duration of the period when calves are with their mother, determines the duration in YJ and YFbirth	180	Expert knowledge
${\phi }_{G}^{-1}$	Duration of gestation	274	Expert knowledge
${\tau }^{*}$	Age before which natural mortality is applied	21	Expert knowledge^a^
${p}_{{A}_{e}}$	Probability to abort when infected in early gestation (0 to 42 days)	0.8	[7, 33, 40]
${p}_{{A}_{m}}$	Probability to abort when infected in mid-gestation (43 to 150 days)	0.25	[7, 34]
${\phi }_{M}^{-1}$	Duration of the protection given by maternal immunity	150	[35]
${\phi }_{T}^{-1}$	Duration of transient infection	7	[33]
${p}_{P}$	Probability of vertical transmission in mid-gestation (43 to 150 days)	0.937	[, 7, 34, 35]
${p}_{{E}_{P}}$	Probability of disease-related mortality, neglecting mortality at birth	0.00189	[8]
${\beta }_{{T}_{w}}$	Transmission rate per transient animal within its group	0.03	[36, 37]^b^
${\beta }_{{P}_{w}}$	Transmission rate per persistent animal within its group	0.5	[37, 38]
${\beta }_{{P}_{b}}$	Transmission rate per persistent animal to other groups	0.01	[39]^b^
${\beta }_{{P}_{n}}$	Transmission rate per persistent animal in geographically neighbouring herds that can access to pasture	0.001	[31]^b^
Radius	Maximum threshold distance (in km) used for defining pathogen transmission through pasture	2	Expert knowledge

All durations are in days.

^a^According to observed data (FCID) most natural mortality occurs in the first weeks of age.

^b^No experiments nor field studies have yet provided an estimation of these parameters. Therefore, we choose values similar to those used in previous modelling studies.

**Figure 3:**
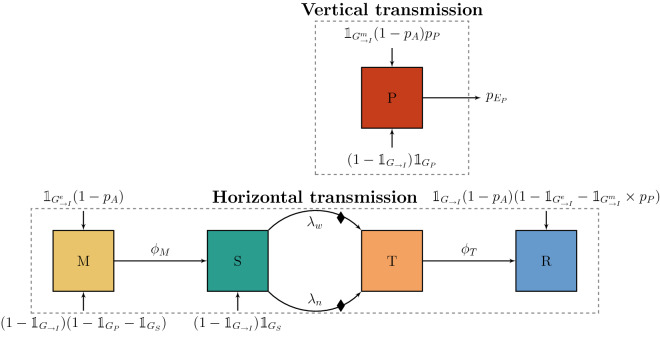
**Health-state dynamics describing vertical and horizontal (within herd or through geographic neighbourhood) transmission.** Arrows represent transitions between health-state compartments. The coefficients on the arrows are either transition rates or transition probabilities. A diamond indicates that on transitioning from S to T, if the animal is G (gestating female), the health-state of the future calf can change or the calf can be aborted. Only births and deaths with a role in infection-related dynamics are represented. See Table 4 and Additional file 1 for parameter definitions.

Further, we considered five mutually exclusive health states: P (persistently infected), M (protected by maternal antibodies), S (susceptible), T (transiently infected), and R (recovered). The schematic for health-state dynamics can be found in Figure 3, where only transitions related to health states are represented. P animals are highly infectious all their life, and can die from the disease with probability ${p}_{{E}_{P}}$.

After ${\phi }_{M}^{-1}$ days, animals in M become susceptible (S). Susceptible animals can become infected either by contact with infected (T or P) animals within their own herd, with a force of infection ${\lambda }_{w}$ (Equation 1), or by contact with P animals of geographically neighbouring herds, with a force of infection ${\lambda }_{n}$ (Equation 2). Such horizontal transmission leads to a transient infection (state T), and possibly to vertical transmission in gestating animals, after which T animals recover (state R). In summary, the three possible ways of transmission are:

- *Horizontal transmission through within-herd contact*, structured by the three groups that determine animals main contacts at the within-herd level: *bred*, *fat*, and *juv* (defined in Additional file 1). Hence, the force of infection at the within-herd level for a susceptible animal is
  1 $${\lambda }_{w} = {{1}_{bred}{\lambda }^{bred} + {1}_{fat}{\lambda }^{fat}+ 1}_{juv}{\lambda }^{juv} ,$$
  which will either be equal to ${\lambda }^{bred}$, ${\lambda }^{fat}$, or ${\lambda }^{juv}$, depending on the group to which the susceptible animal belongs.
- *Horizontal transmission through the geographic neighbourhood*, determined by the force of infection
  2 $${\lambda }_{n} = {1}_{pasture} \times {1}_{bred} \times {\beta }_{{P}_{n}} \times {P}_{n},$$
  which is non-null if the current time is during the pasture period, and the animal is in the *bred* group. ${\beta }_{{P}_{n}}$ is the transmission rate per persistently infected animal in geographically neighbouring herds (cf. Table 4), and ${P}_{n}$ is the number of P animals in geographically neighbouring herds from categories that go to pasture during the pasture period (see Additional file 1 for details).
- *Vertical transmission* (from mother to calf), which occurs if the mother is P, or—with a probability ${p}_{P}$—if the mother gets infected during mid-gestation and the calf is not aborted. See Additional file 1 for details on vertical transmission and maternal protection through antibodies.

Since the FCID does not contain information on life-cycles or health-states, these elements needed to be initialised. See Additional file 1 for a description of this initialisation.

### Epidemiological effect of vaccination

We considered that vaccines can prevent BVD virus vertical transmission if applied before breeding. Then, the probability that a female that was vaccinated before being G produces a P calf, if infected at mid-gestation, is ${p}_{P}^{v} = {p}_{P} \times (1 - {e}_{v})$ during mid-gestation. Other parameters are not modified, so animals can still get infected, be infectious, etc. In particular, if a G female was not vaccinated before breeding, the probability of producing a P calf at mid-gestation remains to be ${p}_{P}$ during this period. In theory, ${e}_{v}$ could be between 0 and 1, yet since available BVD vaccines are quite effective, we set ${e}_{v}$ = 0*.*95.

### Farmers’ dynamic decision-making on vaccination

For the second component of our study, which concerns farmers’ dynamic decision-making on vaccination against BVD, we assumed that vaccination only concerns breeding females, and that vaccination decisions are made following the principles of the mechanism presented in [4], which accounts for farmers’ learning and strategic imitation.

### Decision-mechanism

Algorithm 1 presents the modified decision-making mechanism, where farmers can learn either from one or from all their geographic neighbours and/or selling partners.

**Figure:**
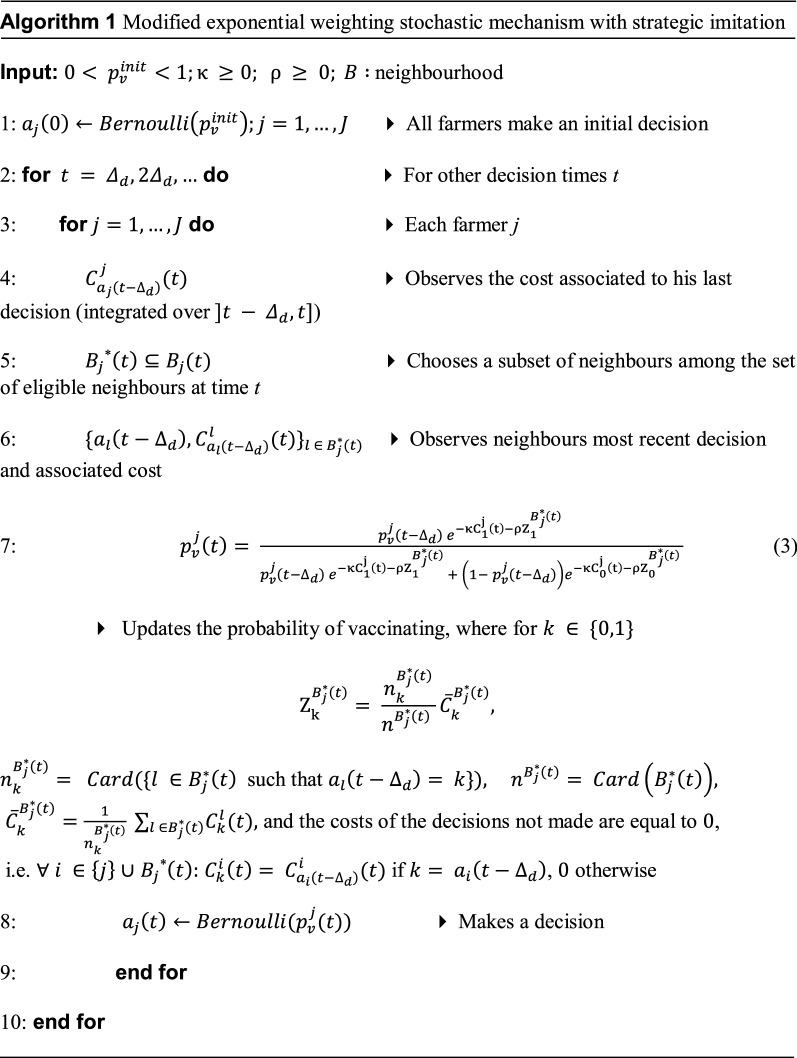

At the first decision time (*t* = 0), each farmer *j* decides to vaccinate or not, according to ${p}_{v}^{init}$, i.e., the initial probability of vaccinating, which is assumed to be equal for all farmers. From the second decision time onwards, each farmer observes the costs associated to the most recent decisions (his own and those of his selected neighbours(s) at that time), updates his probability of vaccinating (as a function of such decisions and costs) and makes a new decision based on this updated probability.

To explain the principles behind the update in the probability that farmer *j* vaccinates at time $t = {\Delta }_{d},2{\Delta }_{d},\dots$, we consider the odds for such an event, ${odds}_{v}^{j}(t),$ which is defined as the ratio between the probability of vaccinating and the probability of not vaccinating. Using Equation 3, the update of the odds is:

4 $${odds}_{v}^{j}\left(t\right)= \frac{{p}_{v}^{j}(t)}{1- {p}_{v}^{j}(t)}= \frac{{p}_{v}^{j}\left(t-{\Delta }_{d}\right) {e}^{-\upkappa {\mathrm{C}}_{1}^{\mathrm{j}}\left(\mathrm{t}\right)-\uprho {\mathrm{Z}}_{1}^{{B}_{j}^{*}\left(t\right)} }}{\left(1- {p}_{v}^{j}\left(t-{\Delta }_{d}\right)\right){e}^{-\upkappa {\mathrm{C}}_{0}^{\mathrm{j}}\left(\mathrm{t}\right)-\uprho {\mathrm{Z}}_{0}^{{B}_{j}^{*}\left(t\right)} }}={odds}_{v}^{j}\left(t-{\Delta }_{d}\right) \times {e}^{(1- 2{a}_{j}\left(t-{\Delta }_{d}\right))\upkappa {C}_{{a}_{j}\left(t-{\Delta }_{d}\right)}^{j}(t)-\uprho {\mathrm{Z}}_{1}^{{B}_{j}^{*}\left(t\right)} +\uprho {\mathrm{Z}}_{0}^{{B}_{j}^{*}\left(t\right)} }.$$

In Equation 4, ${C}_{{a}_{j}\left(t-{\Delta }_{d}\right)}^{j}(t)$ is the cost observed by farmer *j* according to his most recent decision, whose weight is $\kappa$. This weighted cost is multiplied by $1- 2{a}_{j}\left(t-{\Delta }_{d}\right),$ which equals − 1 if farmer *j* vaccinated at time *t,* and equals 1 if he did not. Further, ${\mathrm{Z}}_{1}^{{B}_{j}^{*}\left(t\right)}$ is the mean cost observed by the selected neighbours of *j* that vaccinated, multiplied by the proportion of such neighbours that vaccinated, which is weighted by $\rho$. Similarly, ${\mathrm{Z}}_{0}^{{B}_{j}^{*}\left(t\right)}$ is the mean cost observed by the selected neighbours of *j* who did not vaccinate, multiplied by the proportion of such neighbours who did not vaccinate (also weighted by $\rho$). Hence, the odds of vaccinating are updated proportionally to the previous odds and an exponential function of a weighted sum that depends on the decisions and associated weighted costs of the farmer and his neighbours.

Our decision-mechanism accounts for some simple characteristics of human behaviour. The algorithm itself considers that farmers have a stochastic decision-making process in which they learn from previous experience. In particular, since farmers use their neighbours decisions and observations in their decision-making process, the algorithm accounts for social learning. The exponential weighting scheme integrates cognitive considerations, as farmers quickly increase or decrease their probability of vaccinating according to their observed costs, a feature that has previously been observed in human decision-making [14]. Finally, the update in the probability of vaccinating also accounts for strategic imitation, due to the following three types of situations that can arise according to Equation 4: (i) If both the farmer and all of his selected neighbours vaccinated at the previous decision time, the odds that the farmer vaccinates decrease; (ii) If none of them vaccinated, the odds that the farmer vaccinates increase; and (iii) If they made different decisions, the farmer has information both on vaccinating and not vaccinating (either because he made a decision and all of his observed neighbours made the opposite one, or because he observed neighbours who vaccinated and neighbours who did not). In this case, the decision with the lower weighted cost determines the update. If farmer *j* vaccinated, the weighed cost of vaccinating is $\upkappa {\mathrm{C}}_{1}^{\mathrm{j}}\left(\mathrm{t}\right)+\uprho {\mathrm{Z}}_{1}^{{B}_{j}^{*}\left(t\right)}$ and the weighted cost of not is $\uprho {\mathrm{Z}}_{0}^{{B}_{j}^{*}\left(t\right)}$. Otherwise (i.e. if farmer *j* did not vaccinate), the weighed cost of vaccinating is $\uprho {Z}_{1}^{{B}_{j}^{*}\left(t\right)}$ and the weighted cost of not is $\upkappa {\mathrm{C}}_{0}^{\mathrm{j}}\left(\mathrm{t}\right)+\uprho {\mathrm{Z}}_{0}^{{B}_{j}^{*}\left(t\right)}$. In both cases, if the weighed cost of vaccinating is higher that the weighed cost of not, the odds that *j* vaccinates decrease. If it is lower, they increase. And if they are both equal, the odds are not updated.

We remark that in general, the situation where the odds are not updated can arise either if the weighted observed costs of the farmer and his selected neighbours are zero (if both the farmer and his neighbours vaccinated, this could only occur if $\kappa$ and $\rho$ are null, since the cost of the vaccine doses is positive), or if the weighted observed cost associated with one decision (e.g., vaccinate) is equal to the weighted observed cost associated with the other decision (e.g., not vaccinate). In both cases, it seems reasonable that the probability of vaccinating remains the same, although alternative mechanisms could be formulated.

In summary, Equation 4 can lead to farmers imitating their neighbours [in case (iii)], but can also lead to situations without imitation [in cases (i) and (ii)], where farmers only choose what seems more beneficial depending on their own decisions and those of their neighbours. In the first case, as everyone in the observed neighbourhood vaccinated, there is the underlying idea that the farmer tries to benefit from the vaccination behaviour of his observed neighbours by decreasing the odds of vaccinating (searching to free-ride [15], i.e., benefit from his neighbours’ vaccination without having to bear the economic cost). In the second case, as nobody in the observed neighbourhood vaccinated, the farmer can be considered to be at a high risk, and hence should be more likely to vaccinate. In the third case, the farmer makes a decision by comparing the weighted costs of vaccinating and of not, using his own observations as well as those of his selected neighbours.

Regarding the definition of *B*, the neighbourhood that could influence farmers’ vaccination decisions (lines 5–7 of Algorithm 1), we considered either the geographic neighbourhood ${B}^{g}$ or the "selling" neighbourhood ${B}^{s}$. More specifically:

- *Geographic* neighbourhood: based on geographic proximity and fixed in time. The geographic neighbours are defined as a function of the maximum threshold distance used for defining pathogen transmission through pasture, i.e., 2 km. With this value, the mean number of geographic neighbours is 6.3, and there is a certain heterogeneity in this number across herds (Additional file 1B).
- *Selling* neighbourhood: based on the trade network and therefore time-dependent. The selling neighbours of herd *j* at time *t* are the herds from which *j* purchased animals within the period $]t - {\Delta }_{d},t]$.
  At a given decision time, only herds that are active at that time (i.e., that have animals) can be eligible for being part of the neighbourhood of any other herd. Furthermore, only herds that actually made a decision at time $$t - {\Delta }_{d}$$ (i.e. that had gestating females during the period $$]t - {\Delta }_{d},t]$$) could share this information with their neighbours. Hence, for a given definition of the neighbourhood $$B {(B}^{s}$$ or $${B}^{g}),$$ the set of eligible neighbours of herd *j* at time *t*, $${B}_{j}(t)$$, is the set of such neighbours that made a decision at the previous decision step. That is, neighbours at time *t* that had a non-null quantity of gestating females within the period $$]t - {\Delta }_{d},t].$$ If farmers have no eligible neighbours at a given decision time (i.e., neighbours that made a decision), they decide only as a function of their own observed cost.
  Regarding the way each farmer *j* chooses the subset of neighbours ${B}_{j}$^*^$\left(t\right)\subseteq {B}_{j}(t)$, we considered three options:
- *Random* choosing a neighbour uniformly at random at each decision time among the set of eligible neighbours at that time.
- *Fixed* choosing a neighbour uniformly at random among the eligible neighbours at the decision time ${\Delta }_{d}$, and continuing to observe this neighbour for all the following decision times (hence ${B}_{j}\left(t\right)={B}_{j}({\Delta }_{d})$ for $t={\Delta }_{d},2{\Delta }_{d},\dots$). It is not necessary for the fixed neighbour to have sold animals to the farmer at each period to be eligible (only to have made a decision).
- *All* using information from all eligible neighbours at each decision time.

Finally, we considered an *all–all* option *(*$B={B}^{s}\cup {B}^{g}$*),* where farmers use at each decision time information from all of their trade neighbours and all of their geographic neighbours.

**Figure 4:**
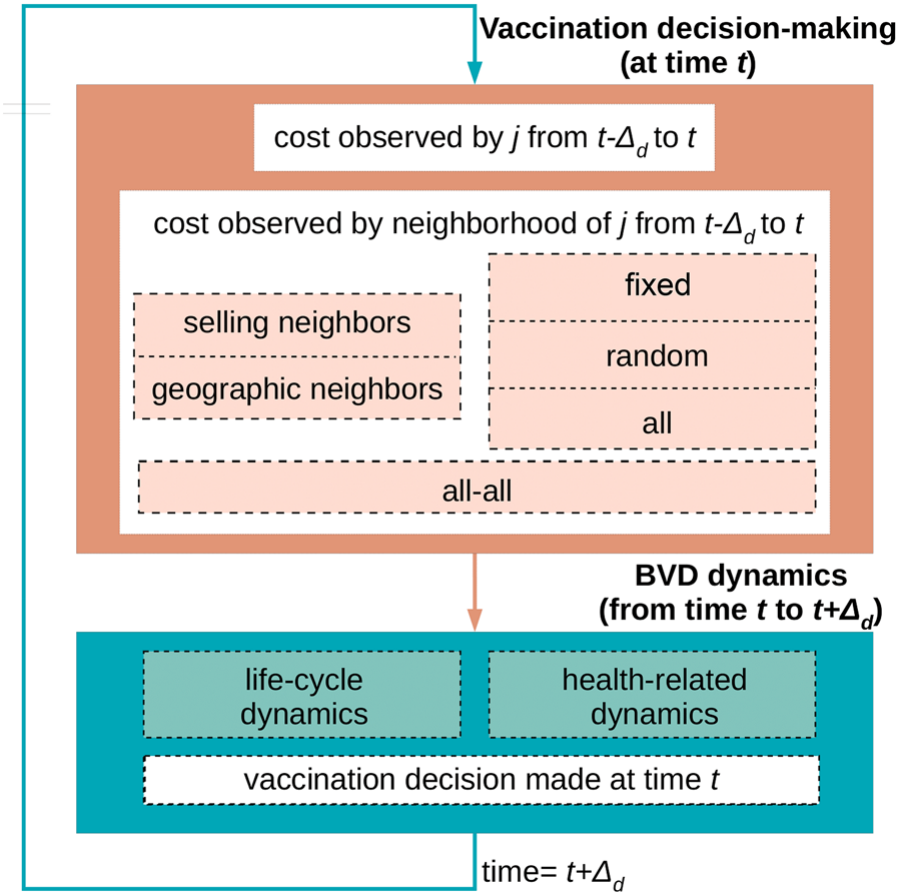
**Schematic of the epidemiological-decision dynamical model for a holding** ***j*** **regarding BVD dynamics and farmers’ vaccination decision-making**.

Figure 4 presents the schematic of the full integrative model for vaccination decision-making in the context of BVD dynamic spread.

### Economic-epidemiological cost

For the definition of the cost function, we modified the one described in [4] in the following way. The cost ${C}_{{a}_{j}\left(t-{\Delta }_{d}\right)}^{j}(t)$ that a farmer *j* observes at time *t*, associated with decision ${a}_{j}\left(t-{\Delta }_{d}\right)$ is computed as:

5 $${C}_{{a}_{j}\left(t-{\Delta }_{d}\right)}^{j}\left(t\right)=\frac{{c}_{v}^{j}\left(t-{\Delta }_{d},t\right)\times {a}_{j}\left(t-{\Delta }_{d}\right) +{c}_{inf}^{j}\left(t-{\Delta }_{d},t\right)}{{N}_{\to G}^{j}\left(t-{\Delta }_{d},t\right)},$$

where,

$${c}_{v}^{j}\left(t-{\Delta }_{d},t\right) = c\_{f}_{v} + [{c\_u}_{v} \times {N}_{\to G}^{j}\left(t-{\Delta }_{d},t\right)],$$

$${c}_{inf}^{j}\left(t-{\Delta }_{d},t\right) = {r}_{1}[{N}_{\to P}^{j}\left(t-{\Delta }_{d},t\right)+{N}_{\to A}^{j}\left(t-{\Delta }_{d},t\right)]+{r}_{2} {N}_{\to T}^{j}\left(t-{\Delta }_{d},t\right).$$

The standardised cost in Equation 5 combines the cost farmers pay to vaccinate breeding females within a given decision period, ${c}_{v}^{j}\left(t-{\Delta }_{d},t\right)$, and the cost of new infections within the same period, ${c}_{inf}^{j}\left(t-{\Delta }_{d},t\right)$. Indeed, the first term in the numerator of Equation 5 is non-zero if vaccination occurred (${a}_{j}(t-{\Delta }_{d}) = 1$). First, ${c}_{v}^{j}\left(t-{\Delta }_{d},t\right)$ considers a fixed cost of applying vaccination per herd, $c\_{f}_{v}$, which could correspond to veterinary fees for vaccination. Second, $c\_{u}_{v}$, is the unitary cost of the vaccine per animal, which is multiplied by the number of vaccinated females for the period, if vaccination is decided on. Putting aside breeding females that did not gestate within the period, this number is supposed to equal ${N}_{\to G}^{j}\left(t-{\Delta }_{d},t\right)$, the number of new gestating females over the period $]t- {\Delta }_{d},t]$. If not applied before the breeding period, vaccination can have adverse effects, but we assume that this is never the case as farmers avoid these problems by vaccinating (if vaccination is adopted) right before the breeding period. Hence, all new gestating females over the period $]t - {\Delta }_{d},t]$ must have been vaccinated before becoming G, if vaccination was decided on for that decision time.

In ${c}_{inf}^{j}(t-{\Delta }_{d},t)$, ${r}_{1}$ is the monetary value of a healthy calf, which is completely lost if this calf is aborted or persistently infected (P). Further, ${r}_{2}$ is the loss associated with a new transient infection of a young animal. Then, ${N}_{\to A}^{j}\left(t-{\Delta }_{d},t\right)$, ${N}_{\to P}^{j}\left(t-{\Delta }_{d},t\right)$ and ${N}_{\to T}^{j}\left(t-{\Delta }_{d},t\right)$ are respectively the cumulative numbers of aborted animals, of P calves, and of young T animals in herd $j$ within the period $]t-{\Delta }_{d},t]$. Indeed, even if vaccines only have a direct effect on preventing vertical transmission, we can consider a broader indirect impact since the presence of P animals in the herd can increase the number of new infections. On the one hand, these new infections may concern gestating females, which can cause more P calves to be born (if infection occurs in mid-gestation) or can lead to abortions (if infection occurs in early or mid-gestation). On the other hand, there can be new infections of young animals (i.e., YJ or YFbirth) which may become transitory infected (T) by contact with P animals. These animals are known to be at higher risk of presenting an enteric and respiratory disease [16].

Finally, to account for differences in the costs of herds, related to the variation in the number of gestating females (G) over the period, the overall cost is standardised by ${N}_{\to G}^{j}\left(t-{\Delta }_{d},t\right)$.

The definition and values of economic and decision-related parameters are summarised in Table 5. For the parameters in ${c}_{v}^{j}(t - {\Delta }_{d},t)$, we arbitrarily supposed $c\_{f}_{v}=50$ euros, as in [4], and $c\_{u}_{v} = 5.11$ euros, the mean cost of one vaccine dose for four different brands of BVD vaccines [13]. For the parameters in ${c}_{inf}^{j}\left(t-{\Delta }_{d},t\right)$, we set *r*_1_ = 800 euros, i.e., roughly the price of a calf of the Charolais breed [17]. The parameter ${r}_{2}$ is somewhat more difficult to set as the precise loss associated to an animal becoming T is harder to calibrate, though it is expected to be much lower than *r*_1_. Nevertheless, the associated overall cost may be high if there are many new T animals. We chose to set ${r}_{2}=0.01\times {r}_{1}=8$€. We note that in [16] a value of $\pounds 3$ was assumed for the cost of an immunocompromised calf, and $\pounds$31 for a calf born with congenital defects, growth retardation, etc. Regarding the values of decision-related parameters, we set ${p}_{v}^{init},$ $\upkappa$ and $\rho$ arbitrarily. Finally, since farmers were assumed to decide whether to vaccinate females before gestation (roughly 9 months), the decision periodicity was set at 1 year, i.e., farmers make a decision for each year on January 1^st^.

**Table 5:** **Economic and decision-related parameters of the model**.

Parameter	Description	Value	Sources
$c\_{f}_{v}$	Fixed cost of applying vaccination per herd	50 €	Arbitrarily chosen^a^
$c\_{u}_{v}$	Unitary cost of the vaccine per animal	5*.*11 €	[13]
${r}_{1}$	Monetary value of a healthy calf	800 €	[17]
${r}_{2}$	Loss associated with a new transient infection of a young animal (YJ or YFbirth)	8 €	Arbitrarily chosen^a^
${p}_{v}^{init}$	Initial probability of vaccinating	0.01	Arbitrarily chosen^a^
$\kappa$	Farmers’ sensitivity to their own observed cost	1	Arbitrarily chosen^a^
$\rho$	Farmers’ sensitivity to the cost observed by their selected neighbours	0.5	Arbitrarily chosen^a^
${\Delta }_{d}$	Decision periodicity	1 year	Expert knowledge

^a^Each parameter was set to a value with a plausible order of magnitude.

### Simulation setting

We explored infection and decision dynamics over a five-year period (January 2009 to December 2013) for $8 \, (=2 \times 3+ 1 + 1)$ scenarios. These scenarios are given by combining the neighbourhood definition (geographic and/or selling) and the way the neighbour is selected (random, fixed, or all). The last one corresponds to an additional baseline scenario (*no_decision*), considered for studying the infection dynamics without the decision component of the model. For all scenarios, the simulation step was set to 7 days, due to the short duration of transient infections.

Given the intrinsic stochasticity and computational cost of the model, we performed 50 runs by scenario. For each, we considered that for a subset of 4% of the herds, 0.2% of their calves (YJ + YFbirth) were initially P. That is, $136 \, (=3416*0.04)$ herds were initially infected with one P calf each. Indeed, the initial total number of calves in the study metapopulation for the year 2009 was 52 513. The 0.2% P calves corresponded to having at least 105 P calves in total, and hence that each positive herd had one $(\approx 105/136)$ P calf. We defined these initial conditions on the basis of information from animal health services (GDS: *groupement de défense sanitaire*) of Saône-et-Loire [18] for the year 2021, which indicated 273 positive calves out of 184 930 tested (i.e., 0.2%), belonging to 109 of the 2900 herds tested; that is, 3.8% (= 109/2900) of the herds had at least one P calf among the tested animals. We note however, that there was no measurement of vaccination practices in the study, which may partly explain such a low prevalence for that first general screening.

The P calf for each initially infected herd was chosen among the YJ or YFbirth beef animals of the selected herd, according to a multinomial distribution, where the probabilities of the P animal being a YJ/YFbirth animal were set proportional to the initial proportion of young beef animals in the chosen herd that were YJ/YFbirth. We remark that only herds that initially had at least one YJ or YFbirth beef animal were eligible to be initially infected, so as to ensure the same number of initially infected P animals and the same initial number of infected herds for all runs.

As decision parameters were arbitrarily chosen, additional numerical settings were considered. To evaluate the impact of the initial probability of vaccinating, we considered a setting where $\kappa = 1$ and ${p}_{v}^{init}$= 0.10. Also, we considered three other settings defined by different values for $\upkappa$: 0.01, 0.1, and 10. In these three settings, the value of ${p}_{v}^{init}$ was kept the same as in the main numerical setting, i.e., ${p}_{v}^{init}=$ 0.01. The value of $\rho$ changed as a function of $\upkappa$ since we kept the ratio $\upkappa /\uprho = 2$ (i.e., we always assumed that farmers put a weight on their own observed cost that was twice the weight they put on observations of their neighbours). And we also considered a scenario without social learning (i.e., farmers only observe their own costs), by setting $\kappa = 1,\rho = 0$. Finally, we evaluated a scenario where decisions relied only on the neighbours’ observed costs [scenario selling-all ($\kappa = 0, \rho = 0.5$)]. That is, in this scenario, farmers did not take into account their own observed costs, but only those of their selling neighbourhood.

## Results

**Figure 5:**
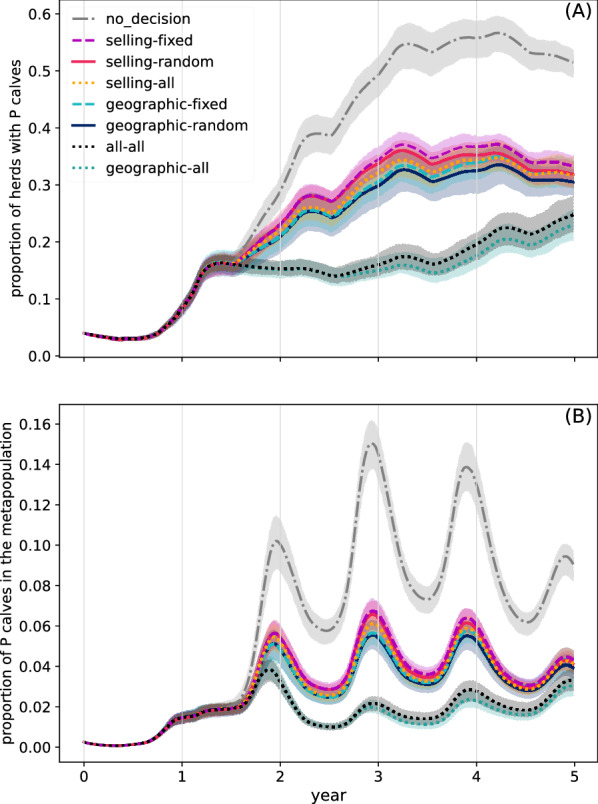
**Dynamics of BVD virus spread in the metapopulation**. Proportion of herds with P calves (**A**) and proportion of P calves (out of all calves) in the metapopulation (**B**). Each colour and line-style represent the neighbourhood (geographic and/or selling) and the way neighbours are selected for observation (random, fixed or all). Gray vertical lines represent decision times. Mean results and 90% confidence bands over 50 runs.

Figure 5 presents infection dynamics, regarding the presence of P calves in the metapopulation, for each of the considered scenarios. Without the vaccination component (no_decision scenario) the pathogen spreads quite rapidly, and at the end of the fourth year, around 55% of herds have at least one P calf. During the fifth year, this mean proportion decreases by roughly 5%. The proportion of P calves (out of all calves) presents a seasonal behaviour most likely due to the birth of P calves, with waves whose peaks are attained roughly at the end of each year, from the second year on. The highest peak (around 0.15) occurs at the end of the third year. The final (at the end of the fifth year) mean proportion of P calves in the metapopulation is roughly 0.09.

**Figure 6:**
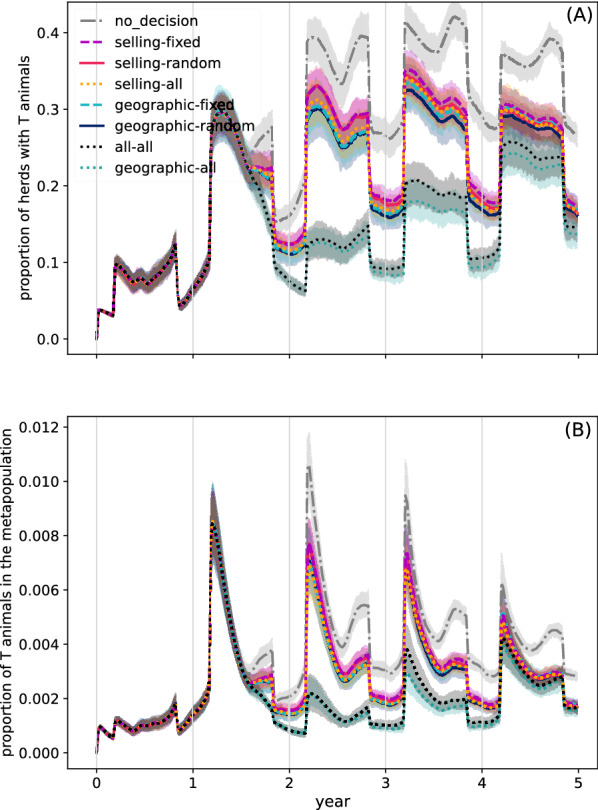
**Dynamics of BVD virus spread in the metapopulation.** Proportion of herds with T animals (**A**) and proportion of T animals in the metapopulation (**B**). Each colour and line-style represent the neighbourhood (geographic and/or selling) and the way neighbours are selected for observation (random, fixed or all). Gray vertical lines represent decision times. Mean results and 90% confidence bands over 50 runs.

Regarding the proportion of herds concerned by the presence of T animals (Figure 6A), it also exhibits a seasonal behaviour consisting of two peaks per year (roughly arriving within the pasture period), with the highest peaks over the five-year period attaining 0.4. The annual dynamics for this quantity between the third and the fifth year are quite similar. The proportion of T animals in the metapopulation, Figure 6B, also shows seasonal peaks over the same periods. The mean proportion is almost 0.01 at the highest peak, attained between the second and third years. The dynamics year by year are very similar, and particularly so in years 3–5.

Regarding the scenarios involving decision-making, we first note that all of these lead to a decreased pathogen spread (P and T herds and animals) when compared to the no_decision scenario (Figures 5 and 6). The effect of a decision made at a given time is observed roughly after 9 months, which corresponds to the moment where calves whose gestation was impacted by the most previous decision are born, i.e., those born within the first 9 months of a year are not concerned by the most recent decision.

The scenarios for which pathogen spread is best controlled are those where farmers take into account all of the information from their geographic neighbourhood, i.e., the geographic-all and all-all scenarios. In these scenarios, the mean proportion of herds with P calves (Figure 5A) reaches 0.16 in the second year (as in all the other scenarios), then remains relatively stable until the middle of the fourth year, where it increases to 0.2. At the end of the fifth year, its level (roughly 0.25) is quite close to those of the other scenarios with the vaccination component. Also, in these two scenarios, the highest peak in the mean proportion of infected calves in the metapopulation (Figure 5B) reaches only 0.04, at the end of the second year. In the following years, this proportion continues to exhibit peaks, which are always lower than in the rest of the scenarios, even if from the end of the fourth year the peaks for these two scenarios increase again. At the end of the fifth year, this proportion is very close to the levels of the other scenarios.

Of the three scenarios that consider all of the information from the respective neighbourhoods, the worst control is obtained when decisions rely only on selling neighbours (selling-all scenario). In particular, the proportion of herds with P calves and the proportion of P calves (Figure 5) are not only higher than in the other two scenarios that consider all information from the defined neighbourhood (i.e., the geographic-all and the all-all scenarios), but also slightly higher than the scenarios where only the information from a single geographic neighbour is taken into account (geographic-random and geographic-fixed scenarios). Similarly, this scenario exhibits dynamics for the proportion of herds with T animals and for the proportion of T animals (Figure 6) that are very similar to those of the scenarios where information from a single selling neighbour is taken into account at each decision time (selling-fixed and selling-random scenarios). In particular, the selling-all scenario is the only scenario among the ones where all of the information from the neighbourhood is used, for which a second peak is observed in the mean proportion of herds with T animals (roughly 0.2) and in the proportion of T animals (almost 0.003), in the second year. Clearly, the performance of the all–all scenario is driven by that of the geographic-all scenario.

Like the selling-all scenario, the group of scenarios where only the information from a single neighbour is taken into account at each decision time can be found in between the no_decision scenario and the two scenarios where the pathogen spread is best controlled (all–all and geographic-all scenarios). With respect to the baseline scenario, in the scenarios of this group the mean proportion of herds with P calves increases but to a lesser degree, attaining roughly 0.3 at the end of the fourth year, then slightly decreases in the fifth year (Figure 5A). For the mean proportion of P calves in the metapopulation, the highest peak drops to 0.06, attained at the end of the third year (Figure 5B). A similar behaviour can be observed for the mean proportion of herds with T animals and the mean proportion of T animals in the metapopulation, whose highest peaks are respectively reduced to roughly 0.35 and 0.008 (Figure 6). The geographic scenarios involving the observation of only one neighbour achieve a better control of pathogen spread when compared to the respective selling scenarios, even if the difference is not as striking as for the comparison between using all of the geographic neighbours or all of the selling neighbours. For both the geographic and selling neighbours, randomly choosing the neighbour exhibits slightly better results than always choosing the same neighbour.

**Figure 7:**
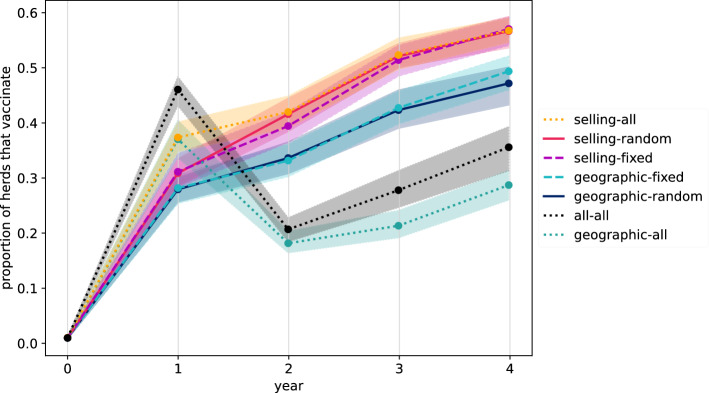
**Dynamics of the proportion of herds that vaccinate.** Each colour and line-style represent the neighbourhood (geographic and/or selling) and the way neighbours are selected for observation (random, fixed or all). Mean results and 90% confidence bands over 50 runs.

Regarding vaccination dynamics, Figure 7 shows that the proportion of herds that vaccinate increases from the initial value (0.01) to 0.28 in the geographic-random and geographic-fixed scenarios, and to 0.31 in the selling-random and selling-fixed scenarios, at the end of the first year. The gap between these two sets of scenarios increases between the first and second years. At the beginning of the fifth year, in the geographic-random and geographic-fixed scenarios, the proportion that vaccinates is around 0.48, while in the respective selling scenarios this proportion attains 0.57.

Regarding the scenarios where all information from the selected neighbourhood is used, both in the geographic-all and in the selling-all scenarios, 37% of herds vaccinate at the second decision time. Meanwhile, in the all-all scenario, this percentage attains roughly 46%. From the third decision time, the proportion that vaccinates in the selling-all scenario is roughly the same as in the other two scenarios that consider selling neighbours (selling-random and selling-fixed scenarios). As for the geographic-all and all-all scenarios, in both this proportion decreases to roughly 0.2 at the beginning of the third year, and then increases to attain 0.28 in the geographic-all scenario, and 0.35 in the all-all scenario, at the beginning of the fifth year.

An examination of the vaccination patterns shows that in scenarios where farmers use only one of their geographic neighbours to decide, the proportion that never vaccinates is higher by roughly 0.08 with respect to scenarios where farmers use only one selling neighbour, and that vaccination patterns whereby herds vaccinate most of the time are also more common in the selling scenarios (Additional file 1C). Finally, Additional file 1D shows that the proportion of herds that never vaccinate is highest in the geographical-all scenario, followed by the all-all scenario. Further, patterns where herds vaccinate most of the time (e.g., 01111 and 00111) are more common in the selling-all scenario. We note also that each of the 32 vaccination patterns are observed in at least one of the scenarios for at least one run.

Results for additional numerical settings can be found fin Additional files 1E–H. These additional experiments show little effect from increasing the initial probability to vaccinate, ${p}_{v}^{init}$, from 0.01 to 0.1 (Additional file 1E). Regarding the impact of varying the values of the two parameters related to farmers’ sensitivity to observed costs ($\kappa$ and $\rho$), as expected, the differences that can be observed between the scenarios using all geographic information and the rest of the scenarios are smaller when the values of such parameters are lower, and more pronounced when the values of $\kappa$ and $\rho$ are higher (Additional file 1F and G). Finally, Additional file 1H shows that considering only each farmer’s own observed cost (no_neighbours $\kappa = 1, \rho = 0$ scenario) does not control pathogen spread as well as when both the information from the farmers and their selling neighbours is taken into account (selling-all $\kappa = 1, \, \rho = 0.5$ scenario). Further, with respect to the latter scenario, the selling-all ($\kappa = 0, \, \rho = 0.5$) scenario (where farmers do not consider their own observed cost) has a roughly similar behaviour, but with differences concerning the proportion of herds that vaccinate.

## Discussion

In this work, the general integrative model proposed in [4] to account for farmers’ dynamic decision-making on vaccination, was adapted to a specific disease: BVD. Modifications partly concerned the economic-epidemiological costs on which farmers base their decisions. Indeed, the fact that our BVD model was structured according to host-heterogeneities (in particular age and life-cycle, and that the vaccine had an effect on vertical transmission, led to a more detailed evaluation of the economic and epidemiological consequences of vaccination decisions. Furthermore, as the BVD virus can spread in different ways (geographic proximity or trade), the initially proposed decision-mechanism was generalised to account for different neighbourhoods and ways of selecting neighbours that could influence farmers’ vaccination decisions. We note that the aim of this article was not to propose a fine-grained characterisation of the BVD virus transmission, but rather to have a transmission model that is sufficiently realistic and relevant to the vaccination decision-making framework we considered.

This work gives insights into the control of the BVD virus spread through voluntary vaccination, by focusing on beef animals in a zone densely populated with Charolais cattle, a beef breeding system for which vaccination has proven to be a cost-effective control measure of interest [13]. The model represents how farmers’ practices can be determined by their own past experiences (vaccinating or not) and by information shared around their neighbourhood regarding other farmers’ experiences. In the model, depending on the decisions and observed costs of a given farmer and his neighbours, the probability that the farmer vaccinates can either increase or decrease.

In our decision-making component, we considered a system of many farmers (or agents) where each farmer faces a decision-making problem under uncertainty [19]. Our objective was to represent farmers’ behaviour when faced to this problem, rather than perfectly solving it (i.e. optimizing their decisions). The problem is related to a large and diverse literature (from economics, psychology, mathematics, artificial intelligence, etc.) dealing with modelling decision-making. Our approach is inspired both by evolutionary game-theory (EGT) [20], which has previously been used in human epidemiology to study vaccination uptake [21, 22], and by multi-armed bandit (MAB) algorithms, classic reinforcement learning techniques used for solving sequential decision-making problems by learning through interacting with the environment [23]. In particular, the update in the odds of vaccinating (Equation. 3) is similar to the update found in the Fermi-Pairwise rule from EGT [24], due to the comparison between the observation(s) of the neighbour(s) and the observation of the farmer. The structure of farmers’ behaviour is similar to a MAB algorithm (particularly to the EXP3 algorithm [25]), as it allows to explicitly account for learning, even though we did not consider an optimization goal. Unlike related works belonging to the social learning literature [26], where individuals also account for the behaviour of others to make their decisions, EGT does not impose an assumption on the rationality of the decision-makers, only that they have a certain heuristic strategy. In particular, our work distances itself from Bayesian learning approaches [27] as agents do not make any belief-based inference on the state of the system. Furthermore, we considered that decisions are taken repeatedly and simultaneously by all agents, i.e. there are not once-in-a-life time decisions that are taken by one individual at a time, as it is the case for informational cascades [28]. Also, we do not consider phenomena such as peer pressure [29], nor study the emergence of herd behaviour [30]. All in all, the phenomena for which our heuristic algorithm allows to simultaneously account are: stochastic behaviour, learning, and the possibility of free-riding or imitation.

Simulations showed that in the two scenarios where farmers observed at each decision time the previous actions and costs of all of their geographic neighbours (geographic-all and selling-all scenarios), the BVD virus did not spread as much as in scenarios where only one geographic neighbour was observed at a time, nor as much as in the scenarios that solely took into account information from trade partners (Figures 5 and 6). In particular, the scenario where all of the information from selling neighbours was accounted for, showed little difference to those where only one neighbour (selling or geographic) was considered at a time. Additionally, for the scenarios where farmers only chose a single neighbour from which to obtain information, the geographic neighbourhood appeared to be slightly better for pathogen control than the selling neighbourhood (Figures 5 and 6). Quite surprisingly, this occurred despite the fact that the proportion of herds that vaccinate when using the geographic neighbourhood was almost always less than that when using the selling neighbourhood (Figure 7). Additionally, the proportion that never vaccinated was higher, and vaccination patterns where herds mostly vaccinate were less common when using the geographic neighbourhood.

Our qualitative results were robust when the values for the parameters regarding farmers’ sensitivity to their own and to their neighbours’ costs were quite high. When these parameters took smaller values, differences among scenarios were less pronounced and, as expected, the dynamics in all scenarios tended to that of the scenario without control. Finally, it was seen that accounting for neighbours’ information can further increase vaccination uptake over and above solely relying on each farmer’s own information (Additional file 1H).

Two main remarks arise from such observations. First, sharing information through the geographic neighbourhood seems to be consistently better for targeting herds that should get vaccinated to reduce BVD virus spread. This is in agreement with previous results on the dominant role of geographic neighbourhood transmission compared to trade-related transmission of the BVD virus [9, 31]. Second, when accounting for the geographic neighbourhood (either solely or together with the selling neighbourhood), using the full information from all neighbours at each decision time seems to have the most important impact on BVD control.

The main limitation of our results concerns the extent to which it is possible to say that the geographic neighbourhood yields better results due to the quality of the information, rather than its quantity. Indeed, as farmers have many geographic neighbours (6.3 on average), almost all farmers had a neighbour from whom to obtain information in the geographic scenarios. However, the majority of farmers have at most one neighbour from whom they purchase animals (0.89 selling neighbours on average. See Additional file 1J), only 60% of them had an eligible neighbour in the selling scenarios, meaning that 40% decided only as a function of their own observed costs. Nevertheless, the scenario where farmers observed all of their selling neighbours exhibited better control of BVD than the scenario where farmers only observed their own costs (Additional file 1H).

The previous remark is related to the form of the probability update given in Equation 3, which in the case where farmers account for all of their neighbours may be more likely to result in situations where each farmer has two types of information at each decision time: costs associated with the type of decision made by the farmer (which includes both the farmer’s cost and the costs observed by neighbours with the same decision), and a cost associated with the other decision (which includes the costs observed by neighbours making the other decision). On the other hand, when each farmer only observes one neighbour at a time, this can only happen if the selected neighbour has made the opposite decision to the one made by the farmer. Otherwise, the farmer has no information associated with the other decision, and therefore the update of the vaccination probability does not occur via a weighted costs comparison. Hence, the update of the vaccination probability is structurally not the same. This could explain why, for the geographic neighbourhood, using all information provided better control than observing only one neighbour at a time (even if the update is made by considering a weighted average cost), while this was not observed for the selling neighbourhood. An interesting perspective on this question would be to explore the shape of the relationship between the distribution of the number of geographic neighbours (which we defined by the maximum radius of transmission through the pasture) and the degree of control achieved for BVD spread.

A second limitation, also found in the original integrative model in [4], is the fact that in the model, farmers perfectly observe costs associated with their own and their neighbours’ decisions. Also, there is the fact that farmers consider in the same way the observations of any of their selected neighbours. In reality, farmers could give greater weight to observations made by their “closer” neighbours, accounting, for instance, for their geographic neighbours proportionally to the distance to their herd, and for their selling neighbours proportionally to the number of animals sold. Through this mechanism, the model could consider an additional strategic behaviour of farmers, as they could better evaluate how the vaccination practices of their neighbours can impact the health status of their own herd.

Additionally, regarding the BVD model itself, we assumed that the metapopulation was initially infected, but that there was no risk of introduction of the virus from outside the metapopulation. As the area modelled is not that large, this assumption could be revisited, and exposure to trade and geographic neighbours from other areas could be accounted for.

We remark that another issue that may be relevant in the general context of controlling infectious diseases with transmission through animal trade concerns the possibility of modifying trade partners, for example, ensuring that trade occurs only between herds of similar risk. Such a type of problem, referred to as *network rewiring* [32], is significantly different from the one we consider here, which involves information sharing and dynamic decisions about whether to adopt a binary health measure. Furthermore, network rewiring can be computationally complex, especially for the setting we consider (a large trade network with internal herd dynamics), and appears to not be as important for BVD, given that the role of the trade network has been shown to be minor compared to that of geographic proximity [31].

Our findings highlight the role that sharing information about health practices through the geographic proximity network can have on controlling BVD spread. This type of result can be useful in the real world for actors such as animal health services, which can play a role in farmers’ practices by advising them or by establishing coordinated actions between them. In France, such services are set up and financed by the farmers themselves, in order to improve health management. Ultimately, it is the farmers who are part of an animal health service (not all farmers are) who decide the focus of the group, and to what extent their actions will be defined by strong restrictions or only recommendations to group members. In this sense, emphasising the role of the geographic proximity can advocate for focusing on certain recommendations or actions, such as advising farmers to avoid grazing animals that may be infected with BVD virus. From a broader perspective, our model could be used as a basis for combining individual and global decision-making (performed by a social planner such as an animal health service). Given the results of our article, it might be relevant, for example, for such a social planner to target efforts on increasing vaccine uptake of geographically disperse herds, so that neighbouring herds start vaccinating based on the strategic imitation mechanism.

In conclusion, we presented in this study a flexible framework for individual decision-making accounting for disease-related features of BVD (pathogen and information transmission through geographic proximity and/or a trade network), while incorporating a specific cost function concerning the economic-epidemiological impact of vaccination against BVD.

## Supplementary Information

**Additional file 1. Details of the BVD model and additional figures.** Details of the BVD model concerning life-cycle and health-related dynamics, as well as their initialisation. Figures on herd size distributions, distribution of the number of neighbours, histograms of vaccination patterns and results for additional scenarios.

## Data Availability

The FCID is available from the French Ministry of Agriculture but restrictions apply. It was used under license for the current study, and so the data are not publicly available with this paper. Data are however available from the authors upon reasonable request, subject to permission from the French Ministry of Agriculture. Code and details on data-based parameter calibration can be found here: https://github.com/CristanchoLina/BVD_farmersdecisions.
