# Supplementary material for: Learning and strategic imitation in modelling farmers’ dynamic decisions on bovine viral diarrhoea vaccination
Source: Vet Res. 2022 Dec 2;53:102. doi: 10.1186/s13567-022-01112-2 (PMC9717531; doi:10.1186/s13567-022-01112-2)
Supplement: Supplementary file 1 — Additional file 1. Details of the BVD model and additional figures. Details of the BVD model concerning life-cycle and health-related dynamics, as well as their initialisation. Figures on herd size distributions, distribution of the number of neighbours, histograms of vaccination patterns and results for additional scenarios. [file 13567_2022_1112_MOESM1_ESM.docx]

**Additional file 1: Details of the BVD model and additional figures**

# Table of contents

**S1 Details of the BVD model**

S1.1 Life-cycle dynamics.

S1.2 Health-state dynamics

*Horizontal transmission through within-herd contact • Horizontal transmission through the geographic neighbourhood • Vertical transmission*

S1.3 Initialization of life-cycle and health-state

**S2 Additional figures**

# S1 Details of the BVD model

**S1.1 Life-cycle dynamics**

Figure 2 in the main document represents life-cycle dynamics. Females not kept for breeding (which is determined using the probability$p_{bred}$) and males (sex is determined using the probability $p_{Female}$) are grouped from birth to weaning in the model compartment YFbirth. Females kept for breeding are grouped from birth to weaning in model compartment YJ. Unweaned calves are considered to be raised together with their dam, thus are in direct contact with bred adult females. These calves can die with probability $p_{E}$, if their age is at most $\tau^{*}$(“calf mortality”). At weaning (after a duration of $\phi_{Y}^{-1}$ days), calves from YFbirth and YJ go to model compartments OFbirth and OJ, respectively, and are not raised with bred females anymore but in separated groups (i.e. only indirect contacts are possible). Animals in OFbirth are culled $\phi_{OFbirth}^{-1}$days after weaning.

Females kept for breeding enter gestation $\phi_{OJ}^{-1}$ days after weaning (transition from OJ to G). Gestation ends at calving or due to infection-related abortion (which occurs with probability $p_{A}$). After calving or abortion, females can be culled with a probability varying with parity. These females are fattened for $\phi_{Fadult}^{-1}$ days (in group Fadult) before being culled. If not culled, females then go to the non-gestating state (NG) for $\phi_{NG}^{-1}$ days, after which a new gestation starts (enter G). In our model, the health-state of a calf is defined as early as gestation begins (when the mother enters compartment G) and is updated if infection occurs during gestation according to the gestation stage (see section S1.2).

The herd-specific parameters (based on the dates of birth, death, calving, and movements in the database) that play a role in the life-cycle dynamics described above are defined in Table 3 of the main document, together with their main descriptive statistics (mean, 10^th^ percentile, median, 90^th^ percentile). Life-cycle parameters that are not herd-specific are defined in Table 4 of the main document. Other life-cycle parameters that are based on herd-based parameters are:

$$\phi_{OJ}^{-1} =\phi_{J}^{-1} - \phi_{Y}^{-1},$$

$${(\phi_{OFbirth}^{Male})}^{-1} ={(\phi_{Fbirth}^{Male})}^{-1}- \phi_{Y}^{-1},$$

$${(\phi_{OFbirth}^{Female})}^{-1} ={(\phi_{Fbirth}^{Female})}^{-1}- \phi_{Y}^{-1},$$

$\phi_{OFbirth}^{-1}= 1_{Male} \times{(\phi_{OFbirth}^{Male})}^{-1} + 1_{Female} \times{(\phi_{OFbirth}^{Female})}^{-1},$

$p_{cull}=1_{P0} \times p_{cull}^{P0} + 1_{P1} \times p_{cull}^{P1}+ 1_{P2} \times p_{cull}^{P2}+ 1_{P3} \times p_{cull}^{P3} + 1_{P4} \times p_{cull}^{P4}+ 1_{P5} \times p_{cull}^{P5} ,$

$cull \sim Bernoulli(p_{cull}).$Defined when a female enters state G,

$\bar{cull}=1-cull$,

$p_{A}= 1_{G_{\to I}^{e}}\times p_{A_{e}}+ 1_{G_{\to I}^{m}}\times p_{A_{m}}$.

In the above, $1_{X}$ represents the indicator function. Then, for $X \in[Male,Female,P0,P1,P2,P3,P4,P5], 1_{X} = 1$ if the animal belongs to compartment $X$, and equals 0 otherwise. Also, $1_{G_{\to I}^{e}}=$1 if the gestating female gets infected during early gestation (0–42 days), and equals 0 otherwise. Similarly, $1_{G_{\to I}^{m}}=$1 if the gestating female gets infected during mid-gestation (43–150 days), and equals 0 otherwise. In the following, we note by $1_{G_{\to I}}$ the indicator function for the infection of a gestating female, regardless of the period in which infection occurs (i.e. $1_{G_{\to I}}=$1 if it gets infected during gestation, and equals 0 otherwise).

# S1.2 Health-state dynamics

## S1.2.1 Horizontal transmission through within-herd contact

Within-herd, animals are assumed to be separated into three groups that determine their main contacts:

- $bred$ = {G, NG, YJ, or YFbirth}: breeding females and their calves (as all beef calves remain with their mother until weaning).
- $fat$ = {Fadult, OFbirth}: fattened animals (all except YFbirth ones, which are found with their mothers).
- $juv$ = {OJ} weaned females that will breed but have not started yet. These animals are neither in direct contact with breeding females and their calves, nor with fattened animals.

Susceptible animals of each group can get infected (enter T) through direct contacts with P or T animals within their own group (infection due to T animals is not neglected since animals are in close contact). Susceptible animals can also become infected through indirect contacts with persistently infected animals located in the other groups. As T animals are not as infectious as P animals, it is unlikely that they contribute to indirect transmission. Hence, the force of infection at the within-herd level for a susceptible animal depends on the group the animal belongs to. That is,

$\lambda_{w}= 1_{bred}\lambda^{bred}+1_{fat}\lambda^{fat}+1_{juv}\lambda^{juv}$*,* (S1)

which will either be equal to $\lambda^{bred}$, $\lambda^{fat}$, or $\lambda^{juv}$, depending on whether the susceptible animal belongs to group $bred$,$fat$, or $juv$, with:

$$\lambda^{bred}= \frac{1}{N^{bred}}\left[ \beta_{T_{w}}T^{bred}+\beta_{P_{w}}P^{bred}+ \beta_{P_{b}}{(P}^{fat}+P^{juv}) \right],$$

$$\lambda^{fat}= \frac{1}{N^{fat}}\left[ \beta_{T_{w}}T^{fat}+\beta_{P_{w}}P^{fat}+ \beta_{P_{b}}{(P}^{bred}+P^{juv}) \right],$$

$$\lambda^{juv}= \frac{1}{N^{juv}}\left[ \beta_{T_{w}}T^{juv}+\beta_{P_{w}}P^{juv}+ \beta_{P_{b}}{(P}^{bred}+P^{fat}) \right],$$

where $T^{x}$*,*$P^{x}$*,* and $N^{x}$ are respectively the number of transiently infected animals, the number of persistently infected animals, and the total number of animals in group $x = \{juv,bred, fat\}.$ Note that frequency-dependency is assumed for within-herd horizontal transmission.

## S1.2.2 Horizontal transmission through the geographic neighbourhood

Infection through geographically neighbouring herds is possible during the pasture period, which takes place every year from March 1^st^ to November 1^st^. Geographically neighbouring herds are defined as those at a maximum distance of two kilometers (expert knowledge). See Additional file 1B for the distribution of the number of geographic neighbours according to different values of the threshold distance (2, 3, 6 km).

All breeding females and their calves (i.e. the $bred$ group), together with females in Fadult, are assumed to go to pasture. The susceptible animals of a given herd can get infected through indirect contacts with P animals belonging to other herds. The infection of Fadult animals during pasture is neglected because there are no gestating females in this category, thus no important consequence can arise from their infection. However, P animals of this group are accounted for to define the force of infection:

$\lambda_{n}= 1_{pasture}\times1_{bred} \times\beta_{P_{n}} \times P_{n},$ (S2)

where $1_{pasture}$ equals 1 if the current time is during the pasture period, and 0 otherwise, while $1_{bred}$ = 1 if the animal is in the $bred$ group (i.e. in G, NG, YFbirth, or YJ), and 0 otherwise. $\beta_{P_{n}}$ is the transmission rate per persistently-infected animal in geographically neighbouring herds (cf. Table 4). Finally, $P_{n}$ is the number of P animals in geographically neighbouring herds from categories that go to pasture during the pasture period, i.e.

$$P_{n} =\left( P_{n}^{NG}+ P_{n}^{G}+ P_{n}^{YFbirth}+P_{n}^{YJ} \right)+ P_{n}^{Fadult} .$$

Note that density-dependency is assumed for horizontal transmission through the geographic neighbourhood.

## S1.2.3 Vertical transmission

Regarding vertical transmission (from mother to calf) and its consequences on the calf to be born, the health-state of a calf is initialized at the beginning of its mother’s gestation, and is updated if the mother gets infected during gestation (Figure 3 in the main document). Let $1_{G_{P}}$ equal 1 if the gestating female is P at calving and 0 otherwise. Similarly, $1_{G_{S}}$ equals 1 if the gestating female is S at calving and 0 otherwise. If the gestating female does not get infected during gestation ($1_{G_{\to I}}$ = 0), the health-state of the newborn calf is P for those born to P mothers ($1_{G_{P}}$ = 1), S for calves born to S mothers ($1_{G_{S}}$ = 1), and M in all other cases. Otherwise, if the gestating female gets infected ($1_{G_{\to I}}$= 1) and the calf is not aborted, the health state of the calf to be born is updated. First, if infection occurs during mid-gestation ($1_{G_{\to I}^{m}}$= 1), there is vertical transmission with probability$p_{P}$, in which case the calf will be P. Second, if the gestating female gets infected during early gestation ($1_{G_{\to I}^{e}}$= 1), the calf will be born with temporal maternal antibodies (i.e. it enters compartment M). In all remaining cases (infection during late gestation, or no vertical transmission following a mid-gestation infection), the calf will be R, i.e. it will be protected from infection during its whole life, as it has long-lasting antibodies.

# S1.3 Initialization of life-cycle and health-state

Regarding the life-cycle, it is initially assigned from available data on each animal (age, sex, etc.) and the parameters of the source herd (if it belongs to the study metapopulation), or of the destination herd (if it does not). Males are assigned to fattening: they are in YFbirth if their age is less than $\phi_{Y}^{-1}$, and OFbirth otherwise. Females can either be assigned to breeding or fattening. If there is information on the next calving date from the FCID, the female is kept for breeding. In this case, it is is considered to be a juvenile if its age is less than $\phi_{J}^{-1}$ (YJ if less than $\phi_{Y}^{-1}$, OJ otherwise), or it can be G or NG (G if the time to the next calving is less than $\phi_{G}^{-1}$, NG otherwise). If the animal is G, the gestation stage is obtained from the time of the next calving. If there is no information on a future calving, the female is assigned to fattening. In the latter case, and if the age of death is known and at most equal to $\tau^{*}$, the female is assigned to YJ or YFbirth uniformly at random. If there is no information on the age of death, or if such an age is over $\tau^{*}$, the female is assigned to a fattening category, depending on its parity and age. If there is no parity information or if the parity is P0, the female can be YFbirth, if its age is less than $\phi_{Y}^{-1}$, and OFbirth otherwise. Finally, if its parity is at least P1, the female is assigned to Fadult. Animals that are not G are assumed to enter their life-cycle at the beginning of the simulation.

Regarding the health-state, the initial health-state of all animals is S, either at the beginning of a simulation, or when entering the metapopulation from outside (i.e. we assume no risk of the virus entering the metapopulation from outside). To study infection dynamics, an initial number of infected animals (P or T) needs to be specified for some herds of the metapopulation. For those herds, the health-state of the specified number of animals is updated from S to either P or T.

# S2 Additional figures

**
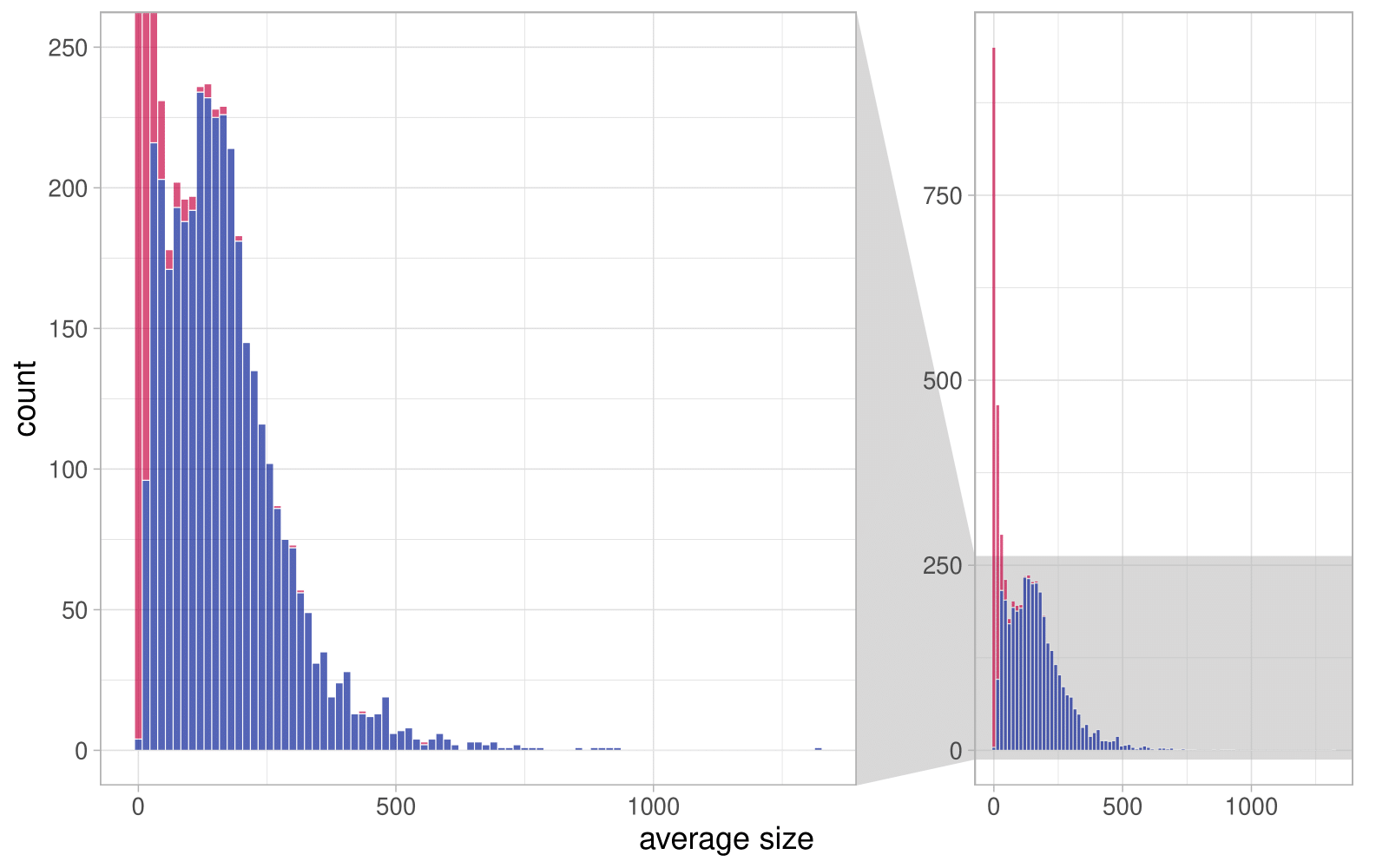
**

**Additional file 1A:** Herd size distributions in Saône-et-Loire French department from data (FCID) for years 2009-2013. Average herd sizes over the five year period. Pink bars represent the distribution for all herds that were active (size *>* 0 at least once within the period), 4978 herds. Blue bars represent the distribution for all herds whose annual average size was at least 30 animals for at least one of the five years, 3416 herds included in the study metapopulation.

**
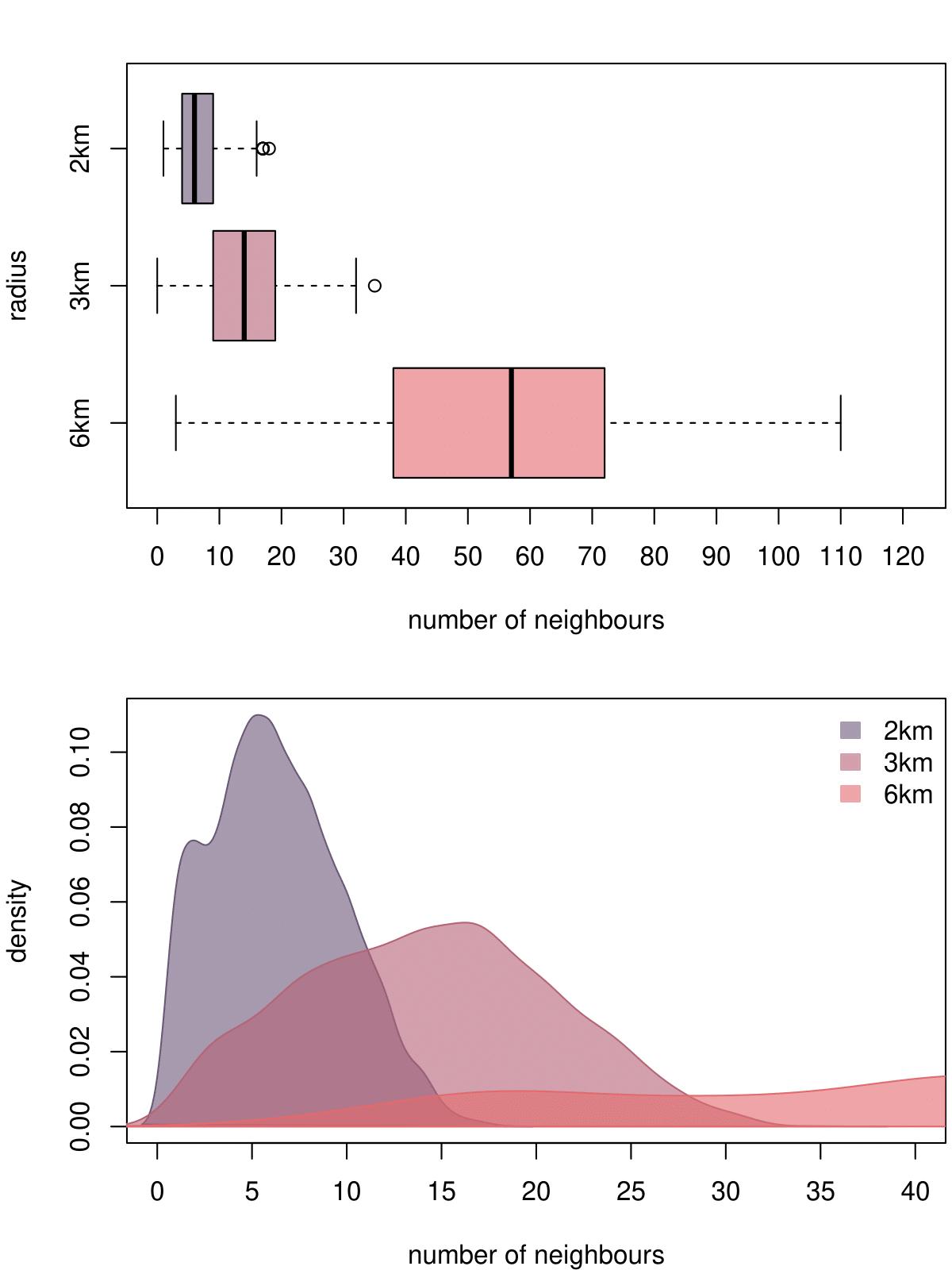
**

**Additional file 1B:** Distribution of the number of geographic neighbours (among the study metapopulation, i.e. 3416 holdings) when the radius to define the geographic neighbourhood is fixed to 6 km, 3 km and 2 km. The bottom figure is a zoom of the distribution (as a density) of the number of geographic neighbours up to 40 neighbours.

**
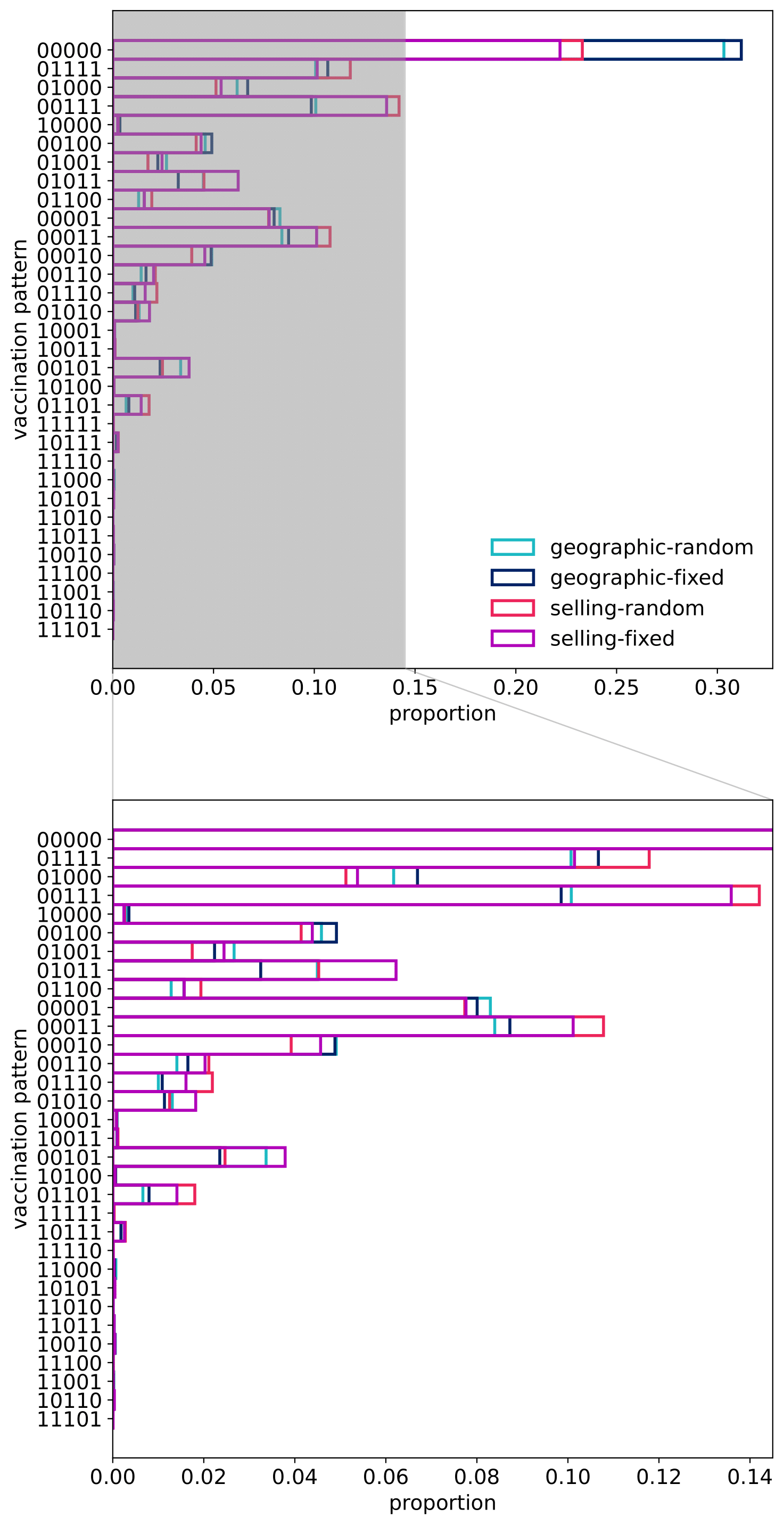
**

**Additional file 1C:** Vaccination patterns for the scenarios where farmers observe the information from only one neighbour (random or fixed) in their neighbourhood (geographic or selling). 0 stands for not vaccinating, while 1 for vaccinating (e.g. the pattern 00010 concerns herds that only vaccinated at the beginning of the fourth year). The bottom figure is a zoom of the top figure that focuses on vaccination patterns that were observed at most in 14% of herds (over runs). Results over 50 runs.

**
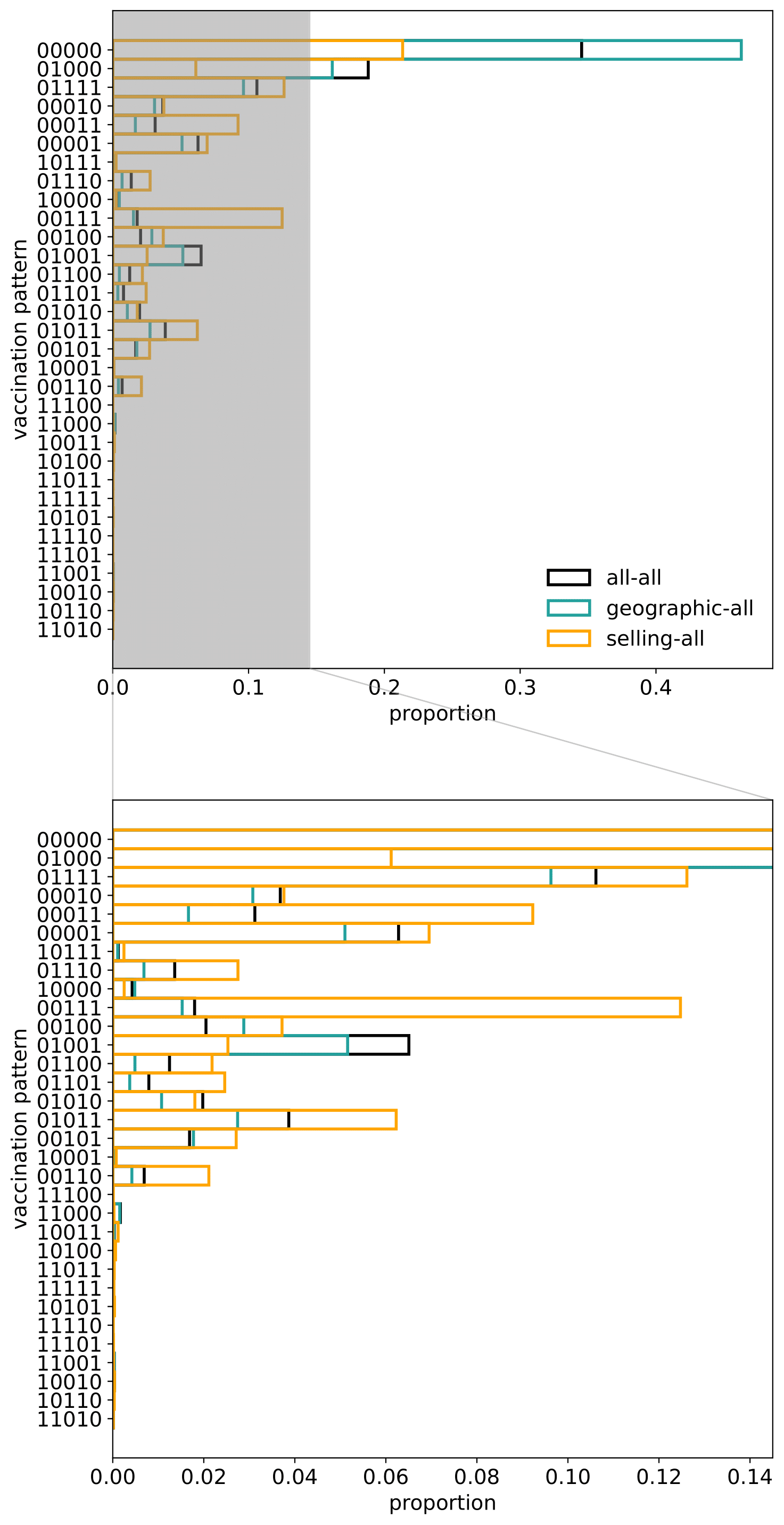
**

**Additional file 1D:** Vaccination patterns for the scenarios where farmers observe the information from all their neighbours in the chosen neighbourhood (geographic and/or selling). 0 stands for not vaccinating, while 1 for vaccinating (e.g. the pattern 00010 concerns herds that only vaccinated at the beginning of the fourth year). The bottom figure is a zoom of the top figure that focuses on vaccination patterns that were observed at most in 14% of herds (over runs). Results for 50 runs.

**
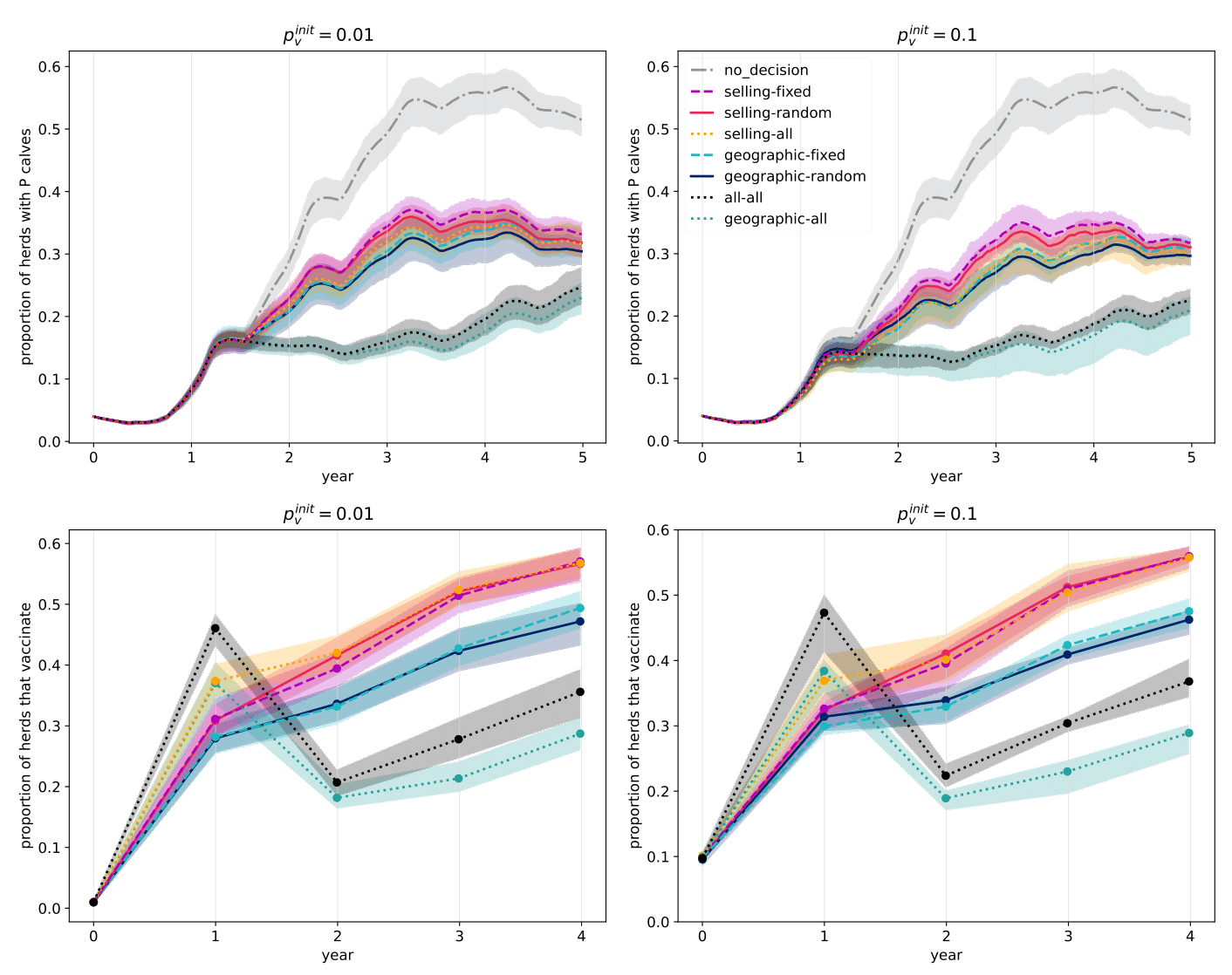
**

**Additional file 1E:** Proportion of herds with P calves and proportion of herds that vaccinate for a different value of $p_{v}^{init}$. In all cases $\kappa$ = 1*,* $\rho$= 0*.*5. Each color and line-style represent the neighbourhood (geographic and/or selling) and the way neighbours are selected for observation (random, fixed or all). Gray vertical lines represent decision times. Mean results and 100% confidence bands over 5 runs. Results for $p_{v}^{init}$= 0*.*01 and for the no_decision scenario over 50 runs are included for comparison.

**
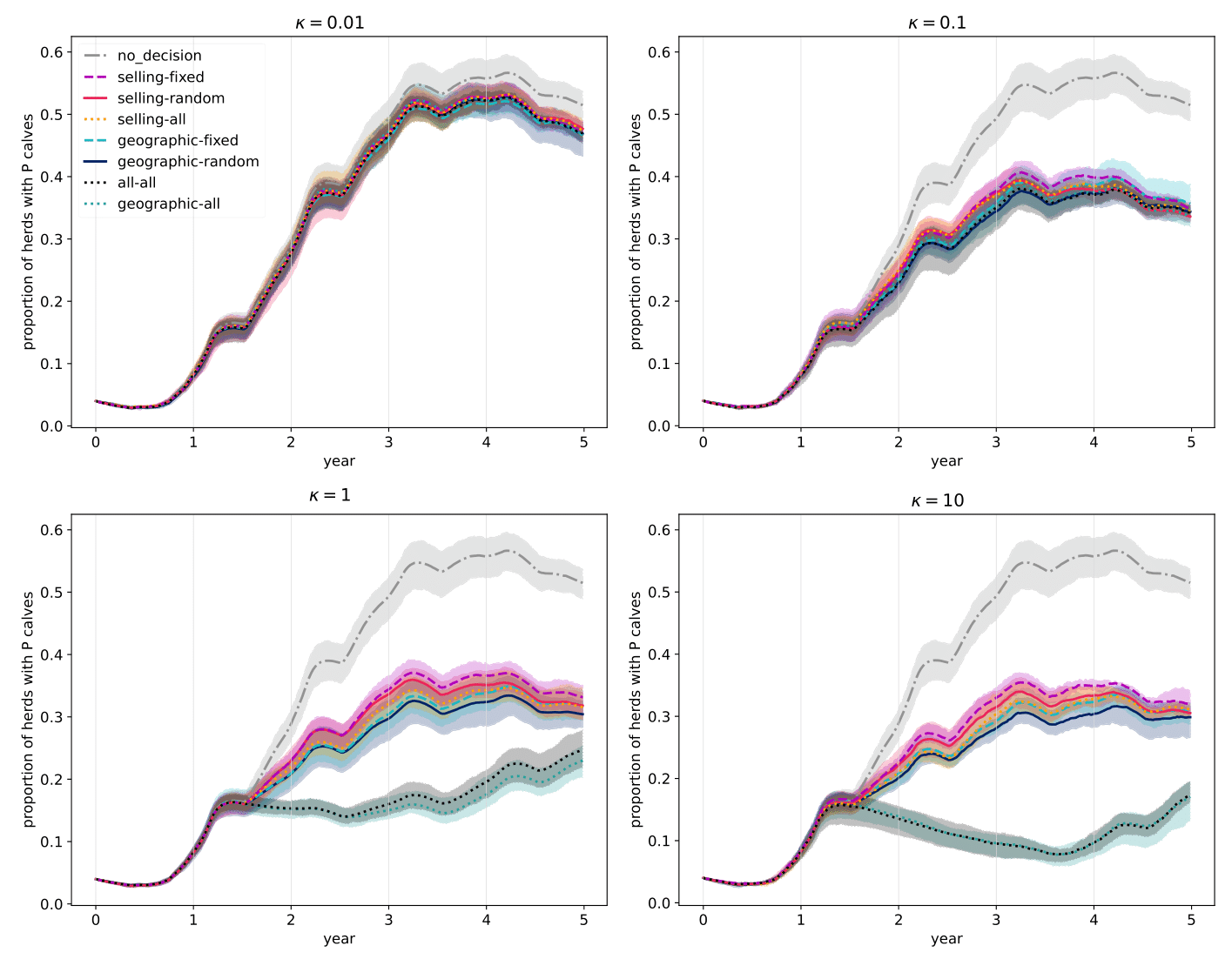
**

**Additional file 1F:** Proportion of herds with P calves for different values of *κ* (where $\rho=\kappa/2$). Each colour and line-style represent the neighbourhood (geographic and/or selling) and the way neighbours are selected for observation (random, fixed or all). Gray vertical lines represent decision times. Mean results and 100% confidence bands over 5 runs. Results for $\kappa$= 1 and for the no_decision scenario over 50 runs are included for comparison.

**
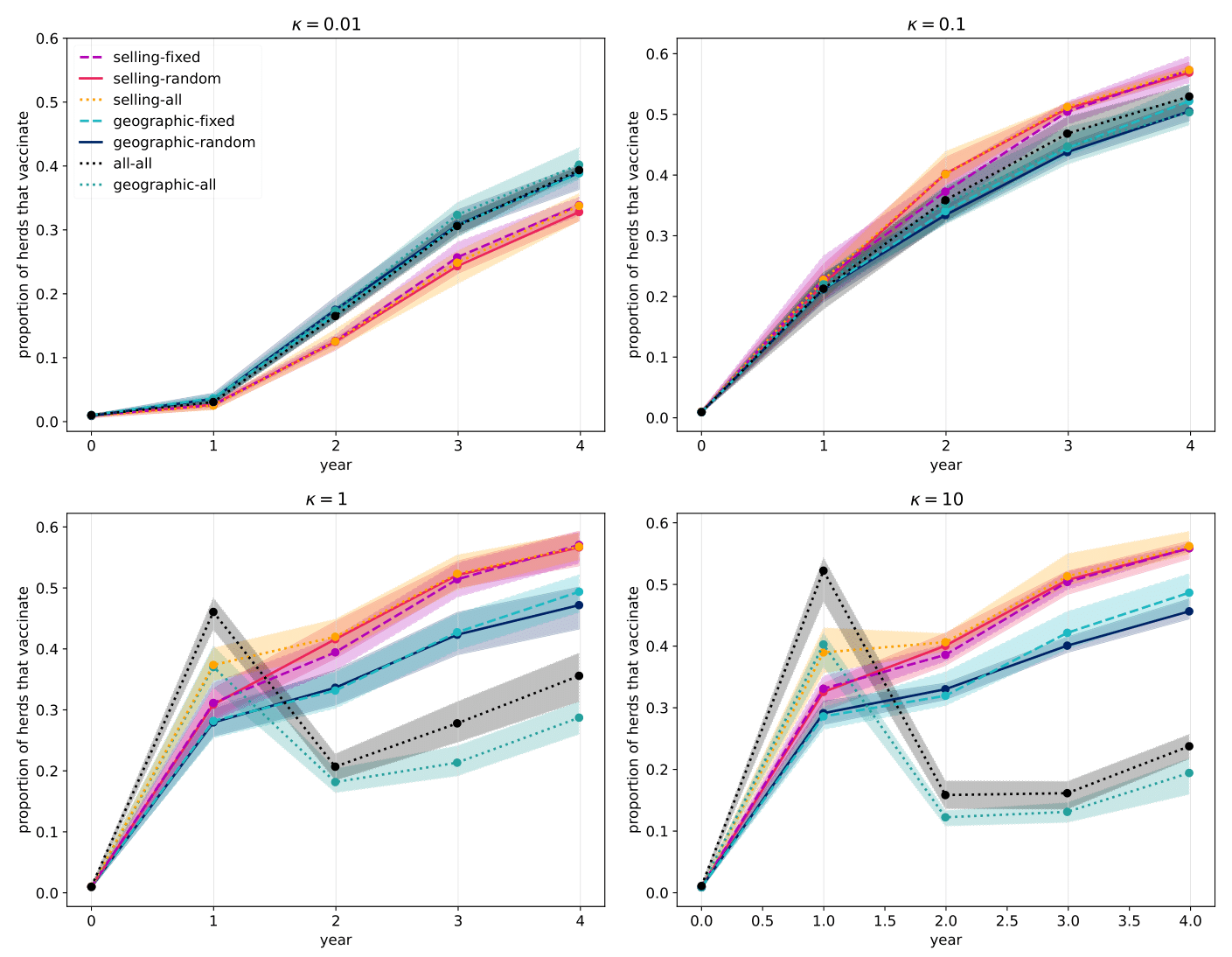
**

**Additional file 1G:** Dynamics of the proportion of herds that vaccinate for different values of $\kappa$ (where $\rho=\kappa/2$). Each colour and line-style represent the neighbourhood (geographic and/or selling) and the way neighbours are selected for observation (random, fixed or all). Mean results and 100% confidence bands over 5 runs. Results for $\kappa$= 1 and for the no_decision scenario over 50 runs are included for comparison.

**
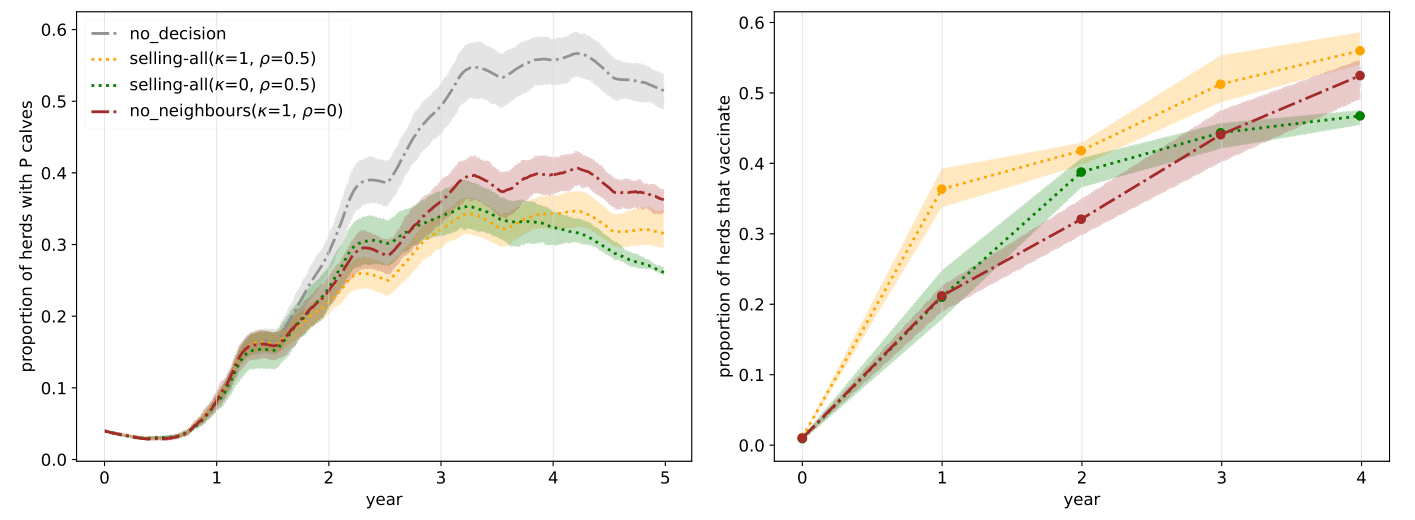
 Additional file 1H:** Proportion of herds with P calves and proportion of herds that vaccinate for different values of $\kappa$ and $\rho$ in the selling-all scenario. Gray vertical lines represent decision times. Mean results and 100% confidence bands over 5 runs. Results for $\kappa$= 1*,* $\rho$= 0*.*5 and for the no_decision scenario over 50 runs are included for comparison.

**
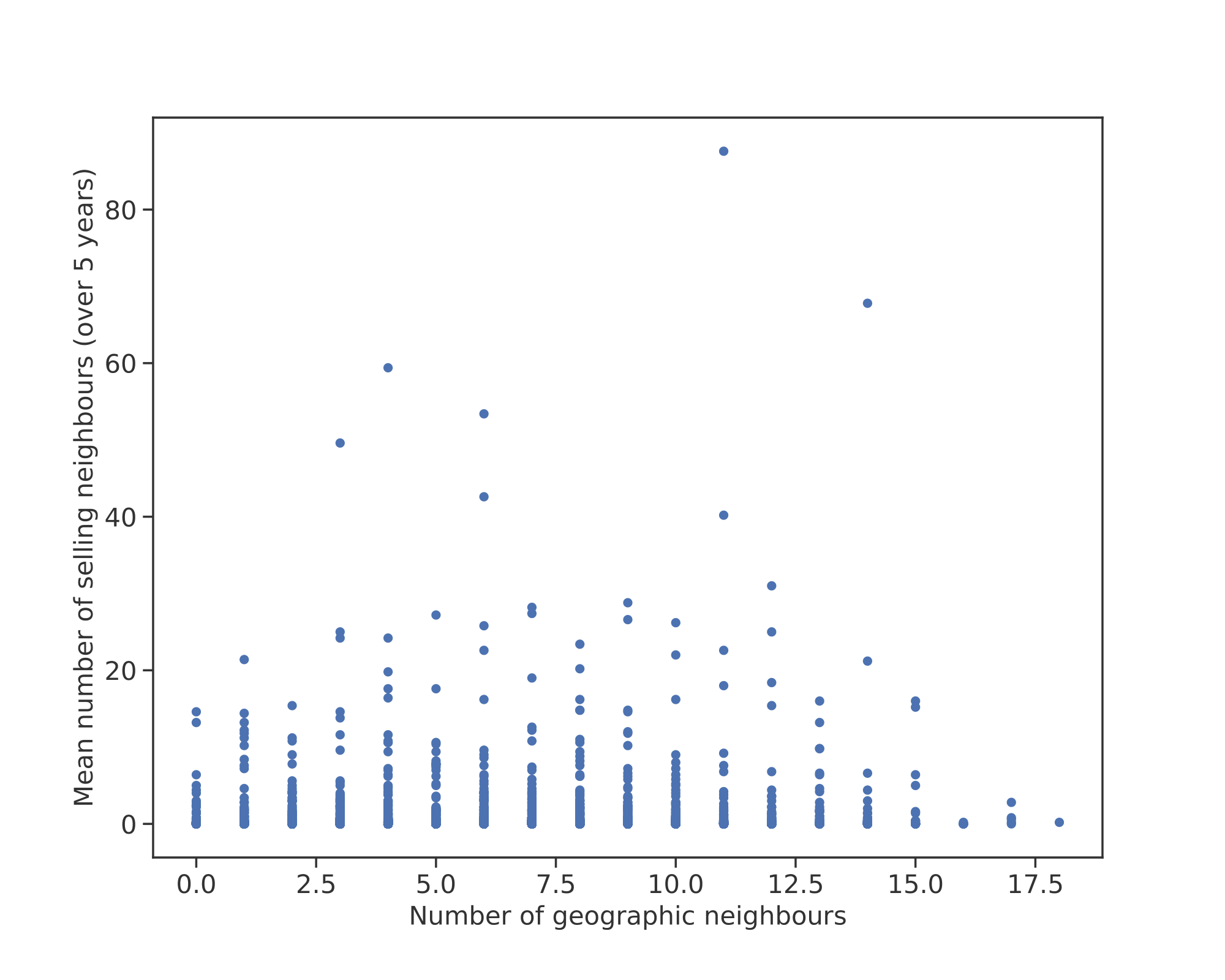
**

**Additional file 1I:** Number of geographic neighbours vs mean number of selling neighbours (over 5 years).
